# A new phenylethyl alkyl amide from the *Ambrostoma quadriimpressum* Motschulsky

**DOI:** 10.3762/bjoc.7.158

**Published:** 2011-09-29

**Authors:** Guolei Zhao, Chao Yang, Bing Li, Wujiong Xia

**Affiliations:** 1 State Key Lab of Urban Water Resource and Environment & the Academy of Fundamental and Interdisciplinary Sciences, Harbin Institute of Technology, Harbin 150080, China

**Keywords:** asymmetric synthesis, beetle, fatty acid amide, isolation

## Abstract

A new phenylethyl alkyl amide, *(*10*R)*-10-hydroxy-*N*-phenethyloctadecanamide (**1**), was isolated from the beetle *Ambrostoma quadriimpressum* Motschulsky. The structure of the amide was determined by NMR and MS. The absolute configuration of compound **1** was confirmed by an asymmetric total synthesis, which was started from L-glutamic acid. The construction of the aliphatic chain was accomplished by the selective protection of the hydroxy groups and two-time implementation of the Wittig olefination reaction.

## Introduction

The leaf beetle *Ambrostoma quadriimpressum* Motschulsky (Coleoptera: Chrysomelidae) is a serious pest of elm that exists primarily in north-eastern China. Adult beetles hibernate over the winter and emerge in the spring when their host plant is most succulent. The developmental stages of beetles are able to defend themselves against predatory attracks, and this defence is associated with chemical defensive compounds.

Insects are able to produce a vast array of biologically active secondary metabolites, which are used for defence purposes or as pheromones. In particular, the chemical defensive phenomenon of the beetle has been observed in Coleoptera [1–2]. Over the last few years, many research efforts have concentrated on chemical communication among beetles [3–6]. Only a limited number of semiochemicals [7–13] are known in leaf beetles. To the best of our knowledge, fatty acid amides from terrestrial insects have not been reported so far. In addition, certain marine organisms [14–15] and mushrooms [16–17] are the only two natural sources of phenylethyl alkyl amides with remarkable activities, such as cytotoxicity to murine leukemia cells (P-388) [14] and protective activity against endoplasmic reticulum stress-dependent cell death [17]. In the current study, we report the isolation of (10*R*)-10-hydroxy-*N*-phenethyloctadecanamide (**1**), a possible chemical defensive compound from the beetle *A. quadriimpressum*, and confirmation of its absolute configuration by total synthesis.

## Results and Discussion

### Isolation of (10*R*)-10-hydroxy-*N*-phenethyloctadecanamide (**1**)

**Scheme 1:**
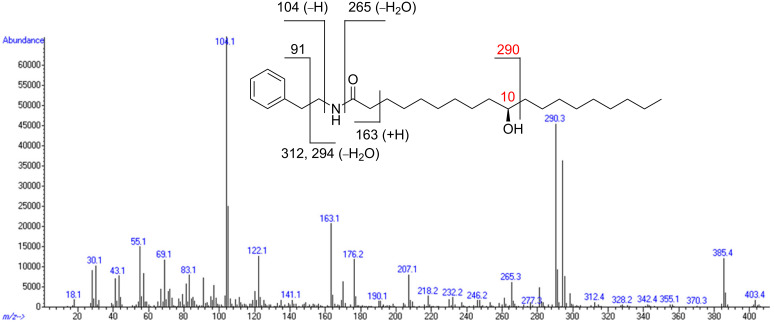
Chemical structure of compound **1** and mass spectral analysis.

Petroleum ether extracts from adult *A. quadriimpressum* were purified repeatedly by means of silica gel column chromatography. Compound **1** was obtained as a white solid. An ion peak at *m/z* 403 and an [M − H_2_O]^+^ peak at *m/z* 385 in the EIMS suggested the presence of an OH group. The molecular formula of **1** was confirmed as C_26_H_45_NO_2_ (*m/z* 404.3529, [M + H]^+^) by HRMS–ESI analysis. The ^1^H NMR spectrum showed aromatic protons [δ 7.33–7.18 (m, 5H)], CH_2_ groups [δ 3.52 (q, *J* = 6.4 Hz, 2H), 2.82 (t, *J* = 6.8 Hz, 2H), 2.11 (t, *J* = 7.2 Hz, 2H), 1.58–1.05 (m, 24H)], an amide proton [δ 5.39 (br s, 1H)], a CH group [δ 3.58 (br m, 1H)] and an Me group [δ 0.88 (t, *J* = 6.8 Hz, 3H)]. The COSY spectrum showed that compound **1** had a coupled proton spin system CH_2_CH_2_NH (δ 5.39, 3.52, 2.82 ppm). Correlations obtained from an HMBC spectrum provided partial evidence to assign a phenylethylamine moiety. The location of the OH group in the aliphatic chain was assigned by EIMS analysis; specifically, evidence was provided by a strong [M − 113]^+^ peak at *m/z* 290 corresponding to the loss of C_8_H_17_ (Scheme 1). Hence, compound **1** was confirmed as 10-hydroxy-*N*-phenethyloctadecanamide. In addition, compound **1** showed positive optical rotation ([α]^20^_D_ +38.5 (*c* 0.13, CHCl_3_)). To confirm the absolute configuration at C_(10)_, an asymmetric total synthesis was performed.

### Synthesis of (10*R*)-10-hydroxy-*N*-phenethyloctadecanamide (**1**)

**Scheme 2:**
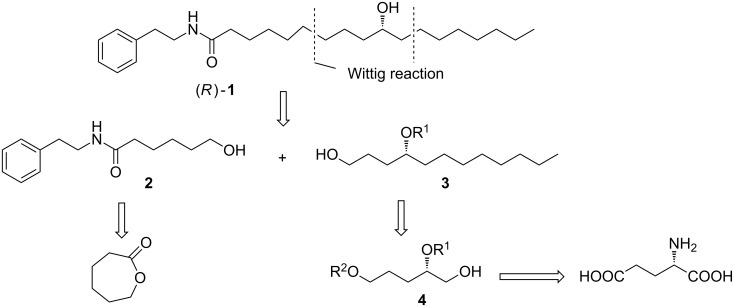
Retrosynthetic analysis of (*R*)-**1**.

The retrosynthetic analysis of compound **1** is outlined in Scheme 2. The target molecule is disconnected into two fragments, **2** and **3**. The intermediate fragment **2** would be readily prepared from the commercially available ε-caprolactone. The crucial fragment **3** would be constructed from the intermediate **4** through Wittig olefination. The polyhydroxy compound **4** would be obtained from L-glutamic acid by selective protection.

**Scheme 3:**
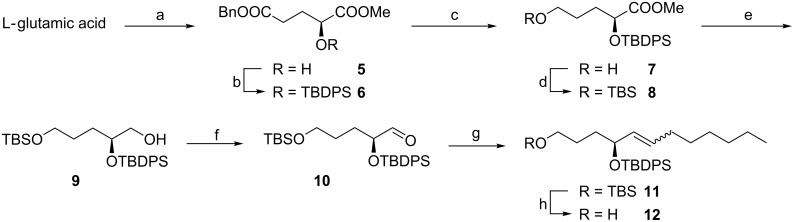
(a) BnOH, H_2_SO_4_; NaNO_2_, AcOH; CH_2_N_2_, 35% in 3 steps. (b) TBDPSCl, imidazole, 100%. (c) Pd/C, H_2_; BH_3_·THF, 95% in 2 steps. (d) TBDMSCl, imidazole, 100%. (e) Dibal-H, 86%. (f) PCC, 94%. (g) C_7_H_15_PPh_3_Br, *n*-BuLi, 75%. (h) PPTS, 98%.

Our synthesis started from the ready available L-glutamic acid, as shown in Scheme 3. On the basis of the preceding literature [18–20], the hydroxy diester **5** was obtained in 35% yield (BnOH, H_2_SO_4_; NaNO_2_, AcOH; CH_2_N_2_). Protection of the secondary alcohol with the TBDPS group gave compound **6** in quantitative yield. The following debenzylation and reduction with BH_3_·THF afforded alcohol **7** in 95% isolated yield. Then primary alcohol **7** was protected again with TBDMSCl to give compound **8** in 100% yield. An initial attempt to synthesize compound **9** by reduction of **8** with LiAlH_4_, however, resulted in the deprotection of the TBDPS group. Alternatively, the alcohol **9** could be obtained by reduction with diisobutylaluminium hydride (Dibal-H) in 86% yield, which was then converted to the corresponding aldehyde **10** by oxidation with PCC. Wittig olefination of aldehyde **10** with *n*-heptylidenetriphenylphosphonium bromide in the presence of *n*-BuLi gave adduct **11** as an (*E*/*Z*)-mixture (*E*/*Z* = 1:5) in 75% yield. Selective removal of the TBS group with pyridinium tosylate gave **12** in 98% yield [21].

**Scheme 4:**
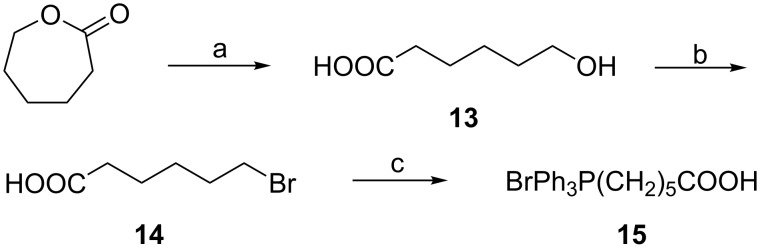
**(**a) 5% NaOH, 100%. (b) PBr_3_, 82%. (c) PPh_3_, 98%.

With the alcohol **12** in hand, our next step was to synthesize the phosphonium salt **15** [22–23] (Scheme 4). Therefore, ε-caprolactone was hydrolyzed with 5% NaOH to give hydroxy acid **13** in quantitative yield, which was reacted with PBr_3_ in CH_2_Cl_2_ to afford compound **14** in 82% yield. Treatment of **14** with PPh_3_ in CH_3_CN gave the phosphonium salt **15** in 98% yield.

**Scheme 5:**
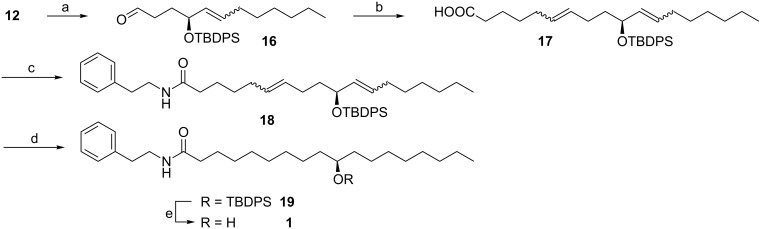
(a) PCC, 96%. (b) LDA, **15**, 72%. (c) DCC, DMAP, phenylethylamine, 80%. (d) Pd/C, H_2_, 100%. (e) TBAF, 93%.

The construction of the aliphatic chain was achieved according to the synthetic plan shown in Scheme 5. Oxidation of the alcohol **12** with PCC afforded the corresponding aldehyde **16**, followed by a Wittig reaction with salt **15** in the presence of two equiv LDA, which afforded the adduct **17** as an (*E*/*Z*)-mixture in 72% yield. The *E*/*Z* isomers of **17** were not separated because the olefin geometry has no effect on the synthesis. Therefore, compound **17** was directly converted to the amide **18** with DCC and DMAP in 80% yield. Reduction of the C=C double bond by hydrogenation with Pd/C gave compound **19** in quantitative yield, which was transferred to the final product (*R*)-**1** in 93% yield by removal of the TBDPS group. Both NMR and EIMS data were in complete agreement with those of the natural product **1**. In addition, the synthetic compound (*R*)-**1** showed positive optical rotation ([α]^20^_D_ +37.2 (*c* 0.85, CHCl_3_)), suggesting that the absolute configuration of natural product **1** is to be assigned (*R*).

## Conclusion

In summary, a new natural product, (10*R*)-10-hydroxy-*N*-phenethyloctadecanamide (**1**) from *Ambrostoma quadriimpressum* Motschulsky was identified and synthesized. Further studies on the biological roles of fatty acid amides are currently being performed by our group.

## Experimental

General Methods: Commercial, spectral-grade solvents were used for the experiments unless otherwise stated. Infrared spectra were recorded on a Perkin–Elmer 1710 Fourier transform spectrometer. Low-resolution mass spectra were obtained from Agilent 7890A-5975C GC–MS by means of electron impact (EI) ionization at 70 eV. ^1^H NMR spectra were obtained at 400 MHz on a Bruker AV-400 instrument. ^13^C NMR spectra were recorded at 100 MHz. High-resolution mass spectra were recorded on an Agilent 1200-6520 Q-TOF electrospray mass spectrometer.

### Isolation of (10*R)*-10-hydroxy-*N*-phenethyloctadecanamide (**1**)

Approximately 3000 adult *A. quadriimpressum* were extracted with petroleum ether. The combined extract was filtered and vacuum dried on a rotary evaporator to give a thick paste (18 g). Flash chromatographic (FC) separation of this extract on a silica gel column with 100%→0% PE/AcOEt gave five fractions. Fraction 4 was further separated into seven fractions on a silica gel column (PE/AcOEt 10:1→1:1). GC–MS analysis showed that fractions 4-4 and 4-5 included derivatives of a fatty acid amide. Fraction 4-5 was further purified by crystallization in diethyl ether to yield compound **1** (3 mg) as a white solid. Mp 107 °C (Et_2_O); [α]^20^_D_ +38.5 (*c* 0.13, CHCl_3_); IR (KBr) *ν*_max_: 3312, 2920, 2846, 1643, 1554 cm^−1^; ^1^H NMR (400 MHz, CDCl_3_) δ 7.32 (t, *J* = 7.6 Hz, 2H), 7.22 (d, *J* = 7.6 Hz, 1H), 7.19 (t, *J* = 7.6 Hz, 2H), 5.39 (br s, 1H), 3.58 (br m, 1H), 3.52 (q, *J =* 6.4 Hz*,* 2H), 2.82 (t, *J* = 6.8 Hz, 2H), 2.11 (t, *J* = 7.2 Hz, 2H), 1.58–1.05 (m, 24H), 0.88 (t, *J* = 6.8 Hz, 3H); ^13^C NMR (100 MHz, CDCl_3_) δ 173.1, 139.0, 128.8, 128.6, 126.5, 72.0, 40.5, 37.5, 37.5, 36.8, 35.7, 31.9, 29.7, 29.6, 29.4, 29.3, 29.2, 25.7, 25.6, 22.7, 14.1; EIMS (*m/z*): 403, 385, 294, 290, 265, 176, 163, 122, 104 (base), 91, 83, 69, 55, 43; HRMS–ESI (*m/z*): [M + H]^+^ calcd for C_26_H_45_NO_2_, 404.3529; found, 404.3529.

## Supporting Information

File 1 Detailed experimental procedures for the synthesis of compound **1**.
