# Supplementary material for: A new phenylethyl alkyl amide from the Ambrostoma quadriimpressum Motschulsky
Source: Beilstein J Org Chem. 2011 Sep 29;7:1342–6. doi: 10.3762/bjoc.7.158 (PMC3201048; doi:10.3762/bjoc.7.158)

Supporting Information

for

A new phenylethyl alkyl amide from the *Ambrostoma quadriimpressum* Motschulsky

Guolei Zhao, Chao Yang, Bing Li and Wujiong Xia*

Address: State Key Lab of Urban Water Resource and Environment & the Academy of Fundamental and Interdisciplinary Sciences, Harbin Institute of Technology, Harbin 150080, China

Email: Wujiong Xia* - xiawj@hit.edu.cn

* Corresponding authors

Detailed experimental procedures for the synthesis of compound 1

Table of Contents

Experimental details S2-S12

^{1}H NMR and ^{13}C NMR spectra of synthetic compounds S13-S30

^{1}H, ^{13}C and 2D NMR spectra of natural product **1** S31-S35

Experimental details

General Methods: Commercial spectral grade solvents were used for experiments unless otherwise stated. Infrared spectra were recorded on a Perkin–Elmer 1710 Fourier transform spectrometer. Low-resolution mass spectra were obtained from Agilent 7890A-5975C GC–MS by means of electron impact (EI) ionization at 70 eV. ^{1}H NMR spectra were obtained at 400 MHz on Bruker AV-400 instrument. ^{13}C NMR spectra were recorded at 100 MHz. High-resolution mass spectra were recorded on an Agilent 1200-6520 Q-TOF electrospray mass spectrometer.

Synthesis of (*S*)-5-benzyl 1-methyl 2-hydroxypentanedioate (**5**)

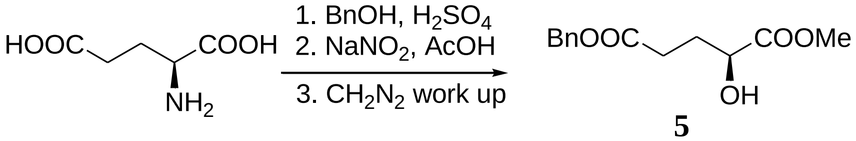

The γ-benzyl ester of glutamic acid (**5**) was prepared according to Rapoport et al. [1-3]. Benzyl alcohol (200 mL, 1.9 mol) was added slowly to a solution of conc. H_2SO_4 (20 mL, 375 mmol) in dry Et_2O (200 mL). The solution was concentrated in vacuo, and glutamic acid (29.6 g, 201 mmol) was added sequentially. The reaction mixture was stirred overnight at r.t., then 95% EtOH (400 mL) and pyridine (100 mL) were added. The mixture was stirred for 1 h in an ice bath. The white precipitate γ-benzylglutamic acid was obtained by filtration, washed with Et_2O, dried at 60 °C for 24 h in vacuo, and was used for next step without further purification (yield 28.6 g, 60%). To a solution of *γ*-benzylglutamic acid (23.8 g, 100 mmol) in H_2O (150 mL) and AcOH (50 mL), a solution of $NaNO_2$ (10.3 g, 149 mmol) in H_2O (100 mL) was added slowly over 4 h. After stirring for 2 h at rt, the reaction mixture was extracted with $CHCl_3$ and isopropanol (v/v = 3:1). The extracts were washed with brine, dried with Na_2SO_4, and concentrated in vacuo to give a colorless oil. The crude product was immediately

esterified with CH_2N_2 in Et_2O, and then concentrated in vacuo. Column chromatography (SiO_2, AcOEt/PE 1:1) of the residue afforded compound **5** (12.4 g, 70%) as a colorless oil. $[\alpha]_D^{20}$ −13.8 (*c* 0.52, $CHCl_3$); IR (film, KBr) ν_{max}: 3487, 2944, 1738, 1455, 1162 cm $^{-1}$; ^{1}H NMR (400 MHz, $CDCl_3$): δ 7.38–7.31 (m, 5H), 5.12 (s, 2H), 4.27–4.22 (m, 1H), 3.77 (s, 3H), 2.98 (t, *J* = 6.4 Hz, 1H), 2.59–2.47 (m, 2H), 2.23–2.16 (m, 1H), 1.99–1.93 (m, 1H); ^{13}C NMR (100 MHz, $CDCl_3$): δ 175.0, 173.0, 135.9, 128.6, 128.3, 128.2, 69.5, 66.4, 52.7, 29.7, 29.3; EIMS (*m/z*): 252, 234, 145, 108, 91(base), 85; HRMS–ESI: Calcd. for $C_{13}H_{16}O_5$: 253.1076 $[M + H]^+$. Found: 253.1080.

Synthesis of (*S*)-5-benzyl 1-methyl 2-(*tert*-butyldiphenylsilyloxy)-pentanedioate **(6)**

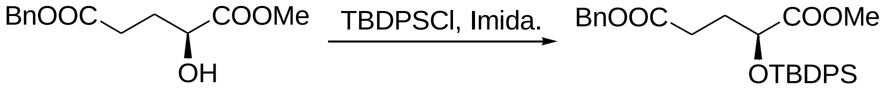

5 **6**

Imidazole (1.36 g, 20 mm) was added sequentially to a solution of **5** (2.52 g, 10 mmol) in dry CH_2Cl_2 (150 mL) at 0 °C. The mixture was stirred for 15 min, and then TBDPSCl (4.12 g, 15 mmol) was added and stirred at rt for 3 h. The reaction mixture was quenched with H_2O and extracted with CH_2Cl_2. The combined extracts were washed with brine, dried (Na_2SO_4) and concentrated in vacuo. Column chromatography (SiO_2, 10% AcOEt in PE) of the residue afforded TBDPS ether (4.9 g, 100%) as a colorless oil. $[\alpha]_D^{20}$ −12.8 (*c* 1.0, $CHCl_3$); IR (film, KBr) ν_{max}: 2945, 2831, 1740, 1455, 1109, 751, 699 cm^{-1}; ^{1}H NMR (400 MHz, $CDCl_3$): δ 7.76–7.71 (m, 4H), 7.69–7.58 (m, 11H), 5.09 (s, 2H), 4.32 (t, *J* = 5.2 Hz, 1H), 3.77 (s, 3H), 2.58 (m, 1H), 2.46 (m, 1H), 2.08 (m, 2H), 1.09 (s, 9H); ^{13}C NMR (100 MHz, $CDCl_3$): δ 172.9, 172.9, 136.0, 135.8, 135.2, 133.1, 132.9, 129.9, 129.8, 128.6, 127.7, 127.6, 71.4, 66.3, 51.5, 30.0, 29.2, 26.9, 19.4.

EIMS (*m/z*): 433, 373, 289, 265, 237, 213, 183, 153, 135, 91(base). HRMS (ESI): Calcd. for $C_{29}H_{34}O_5Si$: 508.2519 $[M + NH_4]^+$. Found: 508.2530.

Synthesis of (*S*)-methyl 2-(*tert*-butyldiphenylsilyloxy)-5-hydroxy pentanoate (**7**)

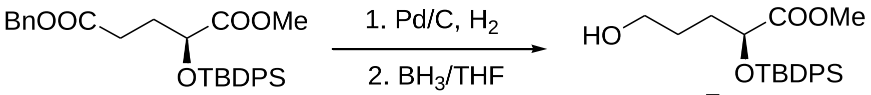

6 **7**

To a solution of **6** (4.9 g, 10 mmol) in MeOH (30 mL), 10% Pd/C (447 mg) was added and the mixture was hydrogenated for 12 h. It was then filtered through a short pad of Celite and the filter cake was washed with MeOH. The filtrate was concentrated in vacuo to give the acid as a colorless oil, which was used without further purification.

To a solution of the above acid in dried THF (30 mL) at –5 °C, BH_3·THF (1.0 M in THF, 11 mL) was added over 10 min. After addition, the reaction mixture was allowed to warm to rt for 3 h. Then it was carefully quenched with MeOH at 0 °C and concentrated in vacuo. Purification of the residue by flash chromatography (SiO_2, AcOEt/PE 1:1) provided the alcohol **7** (3.67 g, 95%) as a colorless oil. $[\alpha]_D^{20}$ –33.2 (*c* 0.93, $CHCl_3$); IR (film, KBr) ν_{max}: 3434, 2952, 1755, 1427, 1111 cm^{-1}; ^1H NMR (400 MHz, $CDCl_3$): δ 7.68–7.63 (m, 4H), 7.44–7.34 (m, 6H), 4.29 (t, *J* = 5.6 Hz, 1H), 3.59 (t, *J* = 6.4 Hz, 2H), 3.49 (s, 3H), 1.84–1.77 (m, 2H), 1.66–1.59 (m, 2H), 1.10 (s, 9H); ^{13}C NMR (100 MHz, $CDCl_3$): δ 173.4, 136.0, 13.8, 133.3, 133.0, 129.9, 129.8, 127.7, 127.5, 72.3, 62.5, 51.5, 31.5, 27.8, 26.9, 19.4; EIMS (*m/z*): 297 (base), 277, 253, 227, 199, 183, 165, 135, 105, 77. HRMS–ESI: Calcd for $C_{22}H_{30}O_4Si$: 387.1992 $[M + H]^+$. Found: 387.1991.

Synthesis of (*S*)-methyl 5-(*tert*-butyldimethylsilyloxy)-2-(*tert*-butyl-diphenylsilyloxy)pentanoate (**8**)

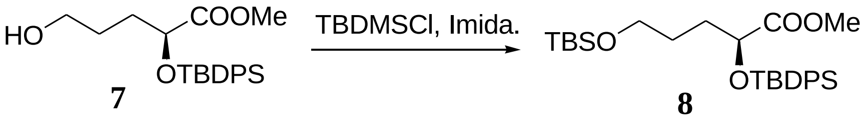

To a solution of **7** in anhydrous CH_2Cl_2 (40 mL) were added imidazole (1.29 g, 19 mmol) and TBSCl (2.15 g, 14.3 mm). After stirring for 5 h at rt, the reaction mixture was quenched with H_2O and extracted with CH_2Cl_2. The extracts were washed with brine, dried (Na_2SO_4), and concentrated in vacuo. Column chromatography (5% EtOAc in PE) of the residue afforded the TBS ether **8** (4.75 g, 100 %) as a colorless oil. $[\alpha]_D^{20}$ −18.3 (*c* 0.52, $CHCl_3$); IR (film, KBr) ν_{max}: 2948, 1757, 1473, 1427, 1257, 1192, 1107 cm^{-1}; ^1H NMR (400 MHz, $CDCl_3$): δ 7.70–7.64 (m, 4H), 7.45–7.36 (m, 6H), 4.27 (t, *J* = 5.6 Hz, 2H), 3.60–3.55 (m, 2H), 3.50 (s, 3H), 1.80–1.76 (m, 2H), 1.65–1.56 (m, 2H), 1.12 (s, 9H), 0.90 (s, 9H), 0.04 (s, 6H); ^{13}C NMR (100 MHz, $CDCl_3$): δ 173.6, 136.0, 135.8, 133.4, 133.2, 129.8, 129.7, 127.6, 127.5, 72.5, 62.8, 51.4, 31.6, 27.9, 26.9, 25.9, 19.4, 18.3, −5.3; EIMS (*m/z*): 485, 443, 415, 355, 309, 283, 213, 183, 135, 91, 75. HRMS–ESI: Calcd for $C_{28}H_{44}O_4Si_2$: 501.2856 $[M + H]^+$. Found: 501.2859.

Synthesis of (*S*)-5-(*tert*-butyldimethylsilyloxy)-2-(*tert*-butyldiphenylsilyloxy) pentan-1-ol **(9)**

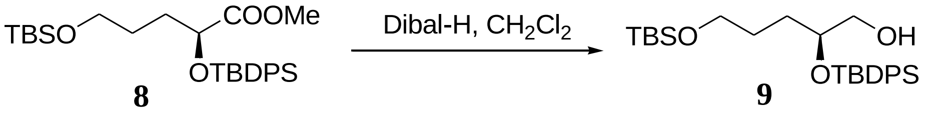

To a solution of **8** (1.1 g, 2.1 mmol) in CH_2Cl_2 at −78 °C Dibal-H (3.0 mL, 1.487 mol/L in toluene, 4.4 mmol) was added. After stirring for 30 min at this temperature, the reaction mixture was then stirred at rt for 2 h. The reaction mixture was then carefully quenched with saturated aqueous NH_4Cl, and diluted with AcOEt, filtered, washed

with brine, dried (Na_2SO_4) and concentrated in vacuo. Purification by flash chromatography (10% AcOEt in PE) gave the product (854 mg, 86%) as a colorless oil. $[\alpha]_D^{20}$ +26.6 (*c* 0.83, $CHCl_3$); IR (film, KBr) ν_{max}: 3452, 2931, 1428, 1111, 702 cm^{-1}; ^1H NMR (400 MHz, $CDCl_3$): δ 7.71–7.67 (m, 4H), 7.46–7.37 (m, 6H), 3.82–3.81 (m, 1H), 3.55–3.40 (m, 4H), 1.87 (t, *J* = 6.4 Hz,1H), 1.65–1.40 (m, 4H), 1.09 (s, 9H), 0.87 (s, 9H), −0.01 (s, 6H); ^{13}C NMR (100 MHz, $CDCl_3$): δ 135.9, 135.7, 134.0, 133.8, 129.8, 127.8, 127.7, 73.9, 65.9, 63.1, 30.0, 28.3, 27.1, 26.0, 19.4, 18.3, −5.3; EIMS (*m/z*): 415, 309, 283, 253, 199, 181, 159, 135. HRMS–ESI: Calcd for $C_{27}H_{45}O_3Si_2$: 473.2907, $[M + H]^+$. Found: 473.2911.

Synthesis of (*S*)-1-(*tert*-butyl(1-(*tert*-butyldimethylsilyloxy)dodec-5-en-4-yloxy)(phenyl)silyl)benzene **(11)**

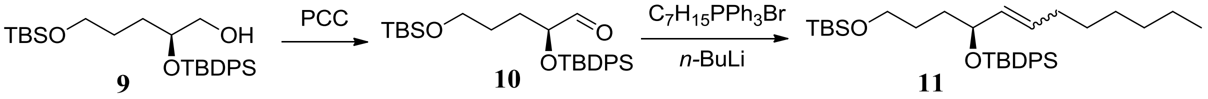

To a solution of PCC (630 mg, 3 mmol) in dry CH_2Cl_2 (30 mL), **9** (709 mg, 1.5 mmol) in CH_2Cl_2 (10 mL) was added slowly and stirred at rt for 2 h. Purification by FC (1% AcOEt in PE) afforded the aldehyde (664 mg, 94%), which was directly used for the next reaction step. To a solution of *n*-heptylidenetriphenylphosphonium bromide (950 mg, 2.1 mmol) in THF (20 mL) at −40 °C, *n*-BuLi (1.0 mL, 2.2 M in hexane, 2.2 mm) was slowly added. The orange colored solution was stirred for 1 h, then aldehyde **10** in THF (5 mL) was added. The mixture was allowed to stir at rt for 12 h and quenched with aqueous NH_4Cl. Ether was added, and the mixture was washed with brine, dried with Na_2SO_4 and concentrated in vacuo. Chromatography of the residue (1% AcOEt in PE) afforded **11** (585 mg, 75%) as a colorless oil. $[\alpha]_D^{20}$ +10.8 (c 1.1, $CHCl_3$); IR (film, KBr) ν_{max}: 2952, 1461, 1427, 1253, 1105, 836 cm^{-1}; ^1H NMR (400 MHz, $CDCl_3$):

δ 7.68–7.64 (m, 4H), 7.41–7.32 (m, 6H), 5.42–5.34 (m, 1H), 5.21–5.18 (m, 1H), 4.43–4.41 (m, 1H), 3.52–3.48 (m, 2H), 2.04–2.00 (m, 2H), 1.58–1.05 (m, 12H), 1.04 (s, 9H), 0.87 (s, 9H), 0.85 (t, J = 7.2 Hz, 3H), 0.05 (s, 6H); ^{13}C NMR (100 MHz, $CDCl_3$): δ 136.0, 135.9, 134.6, 134.5, 132.7, 129.9, 129.4, 129.3, 127.4, 127.3, 69.4, 63.3, 34.7, 31.8, 29.8, 29.0, 28.3, 27.2, 27.02, 25.98, 22.7, 19.3, 18.3, 14.0, −5.3; EIMS (*m/z*): 441, 415, 309, 283, 253, 235, 199, 181, 159, 135, 115, 91, 73; HRMS–ESI: Calcd for $C_{34}H_{56}O_2Si_2$: 575.3717 [M + Na]$^+$. Found: 575.3717.

Synthesis of (*S*)-4-(*tert*-butyldiphenylsilyloxy)dodec-5-en-1-ol (**12**)

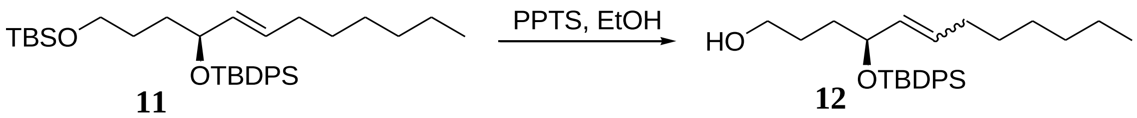

A solution of **11** (420 mg, 0.76 mmol), pyridinium *p*-toluenesulfonate (95 mg, 0.38 mmol) in absolute EtOH (10 mL) was stirred at rt for 24 h. Purification by flash chromatography (CH_2Cl_2) of the residue obtained after removal of the solvent afforded **12** (326 mg, 98%) as a colorless oil. $[\alpha]_D^{20}$ +10.5 (c 0.55, $CHCl_3$); IR (film, KBr) ν_{max}: 3347, 2933, 1471, 1427, 1107, 1056, 821 cm^{-1}; ^{1}H NMR (400 MHz, $CDCl_3$): δ 7.67 (t, J = 8.4 Hz, 4H), 7.44–7.32 (m, 6H), 5.46–5.41 (m, 1H), 5.37–5.18 (m, 1H), 4.49–4.45 (m, 1H), 3.55–3.51 (m, 2H), 1.87–1.49 (m, 6H), 1.25–1.05 (m, 8H), 1.04 (s, 9H), 0.85 (t, J = 7.2 Hz, 3H); ^{13}C NMR (100 MHz, $CDCl_3$): δ 136.1, 136.0, 134.4, 134.1, 132.3, 130.2, 129.6, 129.5, 127.5, 127.3, 69.3, 63.0, 34.7, 31.7, 29.4, 29.0, 28.1, 27.6, 27.0, 22.6, 19.3, 14.0; EIMS (*m/z*): 381, 339, 303, 269, 254, 199, 181, 165, 139, 123, 81; HRMS–ESI: Calcd for $C_{28}H_{42}O_2Si$: 461.2852 [M + Na]$^+$. Found: 461.2853.

Synthesis of (5-carboxypentyl)triphenylphosphonium bromide (**15**)

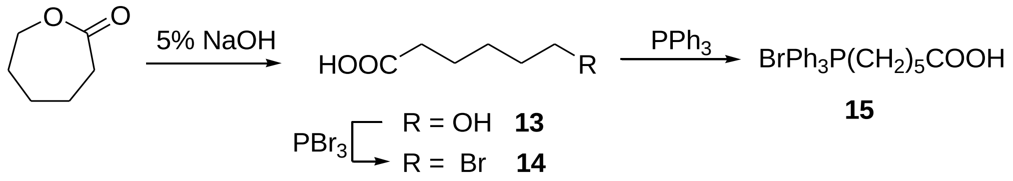

A solution of ε-caprolactone (1.14 g, 10 mmol) in 5% NaOH (50 mL) was stirred at rt for 12 h. The solution was cooled in an ice bath and acidified with 10% HCl. The aqueous phase was extracted with Et_2O three times. The extracts were washed with brine, dried (Na_2SO_4), concentrated in vacuo to give crude product **13** (1.34 g, 100%), which was directly used for next step. To a solution of **13** (1.34 g, 10 mmol) in CH_2Cl_2 (30 mL), PBr_3 (0.8 mL,8.4 mmol) in CH_2Cl_2 (10 mL) was added dropwise at 0 °C. The reaction mixture was warmed to 40 °C and stirred for 5 h. The mixture was cooled to rt, then poured into ice water and extracted with CH_2Cl_2 (50 mL). The extracts were washed (brine), dried and concentrated in vacuo to afford crude compound **14** (1.6 g, 82%). A solution of **14** and PPh_3 in CH_3CN was refluxed under N_2 for 12 h. After removal of the solvent, the residue was washed with Et_2O to give **15** as a white solid (3.6 g, 98%) [4,5]. ^{1}H NMR (400 MHz, D_2O): δ 7.71–7.53 (m, 15H), 3.18–3.10 (m, 2H), 2.17 (t, J = 7.2 Hz, 3H), 1.57–1.36 (m, 6H); ^{13}C NMR (100 MHz, D_2O): δ 178.5, 134.9, 133.5, 133.4, 130.0, 128.9, 118.5, 117.7, 33.3, 29.2, 29.0, 23.4, 21.4, 21.3, 20.9.

Synthesis of (*S*)-10-(*tert*-butyldiphenylsilyloxy)octadeca-6,11-dienoic acid (**17**)

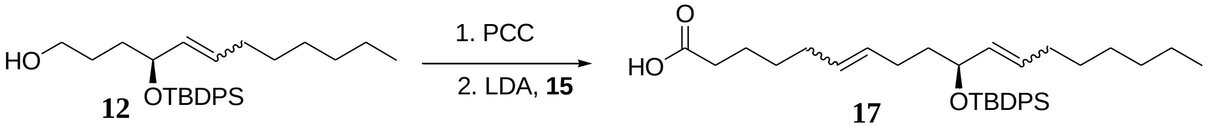

To a solution of PCC (210 mg, 1 mmol) in dry CH_2Cl_2 (10 mL), **12** (230 mg, 0.52 mmol) in CH_2Cl_2 (2 mL) was slowly added and stirred at rt for 4 h. Purification by

flash chromatography (5% AcOEt in PE) afforded the aldehyde (220 mg, 96%), which was used for the next reaction step without further purification.

A stirred solution of diisopropylamine (0.29 mL, 2 mmol) in dry THF (2 mL) was treated under N_2 at −30 °C with *n*-butyllithium (0.91 mL, 2.2 M in hexane). The solution was stirred at −30 °C for 30 min and was added to a suspension of **15** (456 mg, 1 mmol) in dry THF (20 mL). The blood-red solution was stirred for 30 min at rt and treated dropwise with a solution of the above aldehyde in dry THF (2 mL). The mixture was stirred for 3 h. An ice-cold 10% solution of $NaHSO_4$ (20 mL) was added, and the aqueous phase was extracted with AcOEt. The extracts were washed with brine, dried (Na_2SO_4) and concentrated in vacuo. Column chromatography (50% AcOEt in PE) of the residue afforded acid **17** (192 mg, 72%) as a colorless oil. $[\alpha]_D^{20}$ +8.7 (c 0.69, $CHCl_3$); IR (film, KBr) ν_{max}: 3005, 2925, 2853, 1711, 1461, 1427, 1289, 1069, 1109, 823 cm^{-1}; ^1H NMR (400 MHz, $CDCl_3$): δ 7.67 (t, J = 8.0 Hz, 4H), 7.44–7.32 (m, 6H), 5.42–5.36 (m, 1H), 5.32–5.17 (m, 3H), 4.47–4.43 (m, 1H), 2.33 (t, J = 7.6 Hz, 2H), 1.97–1.92 (m, 4H), 1.62–1.05 (m, 16H), 1.03 (s, 9H), 0.85 (t, J = 7.2 Hz, 3H); ^{13}C NMR (100 MHz, $CDCl_3$): δ 178.6, 136.1, 136.0, 134.6, 134.4, 132.6, 130.2, 130.1, 129.5, 129.5, 129.4, 127.4, 127.3, 69.4, 38.5, 33.7, 31.7, 29.4, 29.0, 29.0, 27.6, 27.1, 27.0, 24.3, 22.9, 22.6, 19.3, 14.1; HRMS–ESI: Calcd for $C_{34}H_{50}O_3Si$: 533.3451 $[M - H]^-$. Found: 533.3453.

Synthesis of (*S*)-10-(*tert*-butyldiphenylsilyloxy)-*N*-phenethyloctadeca-6,11-dienamide (**18**)

17

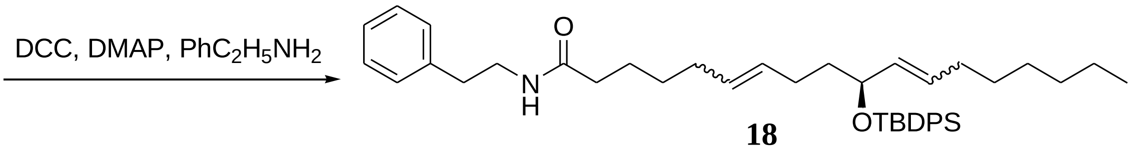

18

A solution of **17** (150 mg, 0.28 mmol), 2-phenylethylamine (34 mg, 0.28 mmol), DCC (58 mg, 0.28 mmol) and DMAP (20 mg) in CH_2Cl_2 (10mL) was stirred for 24 h. The precipitated solid was removed by filtration, the filtrate washed with aqueous HCl (2 N), water and brine, and dried (Na_2SO_4). Solvent removal followed by column chromatography of the residue (10% AcOEt in PE) gave the amide (143 mg, 80%) as a colorless oil. $[\alpha]_D^{20}$ +9.9 (*c* 0.39, $CHCl_3$); ^{1}H NMR (400 MHz, $CDCl_3$): δ 7.67 (t, *J* = 6.4 Hz, 4H), 7.40–7.17 (m, 11H), 5.45–5.37 (m, 2H), 5.26–5.21 (m, 3H), 4.43 (m, 1H), 3.51 (q, *J* = 6.4 Hz, 2H), 2.80 (t, *J* = 7.2 Hz, 2H), 2.09 (t, *J* = 7.6 Hz, 2H), 1.97–1.92 (m, 4H), 1.61–1.05 (m, 16H), 1.03 (s, 9H), 0.85 (t, *J* = 7.2 Hz, 3H); ^{13}C NMR (100 MHz, $CDCl_3$): δ 172.9, 138.9, 136.0, 135.9, 134.6, 134.4, 132.6, 130.2, 129.9, 129.5, 129.4, 129.3, 128.8, 128.7, 127.5, 127.3, 126.5, 69.4, 40.5, 38.6, 36.7, 35.8, 31.7, 29.4, 29.3, 29.0, 27.6, 27.1, 27.0, 26.9, 25.4, 22.9, 22.6, 19.3, 14.1; HRMS–ESI: Calcd for $C_{42}H_{59}NO_2Si$: 636.4237 [M - H]$^-$. Found: 636.4208.

Synthesis of (*R*)-10-(*tert*-butyldiphenylsilyloxy)-*N*-phenethyloctadecan-amide (**19**)

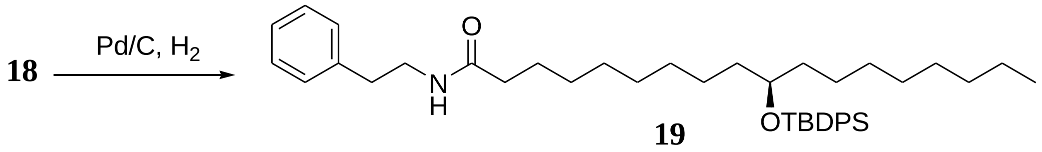

To a solution of the amide **18** (50 mg, 0.078 mmol) in MeOH (20 mL), 10% Pd/C (10 mg) was added and the mixture was hydrogenated for 24 h. It was then filtered through a short pad of Celite and the filter cake was washed with MeOH. The filtrate were concentrated in vacuo to give the product (50 mg, 100%) as a colorless oil. ^{1}H NMR (400 MHz, $CDCl_3$): *δ* 7.67 (d, *J* = 6.8 Hz, 4H), 7.42–7.18 (m, 11H), 5.46 (br s, 1H), 3.70 (m, 1H), 3.51 (q, *J* = 6.4 Hz, 2H), 2.81 (t, *J* = 7.2 Hz, 2H), 2.10 (t, *J* = 7.6 Hz, 2H), 1.58–1.05 (m, 24H), 1.03 (s, 9H), 0.87 (t, *J* = 6.8 Hz, 3H). ^{13}C NMR (100 MHz, $CDCl_3$): *δ* 173.2, 139.0, 136.0, 134.9, 129.4, 128.8, 128.6, 127.4, 126.5, 73.3, 40.5, 36.9, 36.4, 31.9, 29.7, 29.7, 29.5, 29.4, 29.3, 29.3, 27.1, 25.8, 24.9, 22.7, 19.4, 14.1; HRMS–ESI: Calcd for $C_{42}H_{63}NO_2Si$: 640.4550 $[M - H]^-$. Found: 640.4527.

Synthesis of (*R*)-10-hydroxy-*N*-phenethyloctadecanamide (**1**)

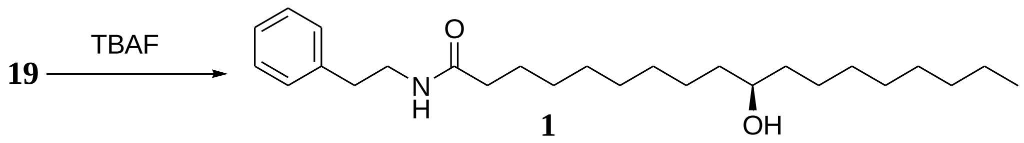

A solution of **19** (50 mg, 0.078 mmol) and TBAF (42 mg, 0.16 mmol) in THF (10 mL) was stirred for 24 h. After removal of the solvent, the residue was purified by flash chromatography (30 % EtOAc in PE) to afford (*R*)-**1** (29 mg, 93%) as a white solid. Mp 106–107 °C; $[\alpha]_D^{20}$ +37.2 (*c* 0.85, $CHCl_3$); IR (KBr) ν_{max}: 3312, 2920, 2846, 1643, 1554 cm^{-1}; ^{1}H NMR (400 MHz, $CDCl_3$): *δ* 7.32 (t, *J* = 7.6 Hz, 2H), 7.22 (d, *J* = 7.6 Hz, 1H), 7.19 (t, *J* = 7.6 Hz, 2H), 5.39 (br s, 1H), 3.58 (br m, 1H), 3.52 (q, *J = 6.4 Hz,* 2H),

2.82 (t, J = 6.8 Hz, 2H), 2.11 (t, J = 7.2 Hz, 2H), 1.58–1.05 (m, 24H), 0.88 (t, J = 6.8 Hz, 3H); ^{13}C NMR (100 MHz, $CDCl_3$): δ 173.1, 139.0, 128.8, 128.6, 126.5, 72.0, 40.5, 37.5, 37.5, 36.8, 35.7, 31.9, 29.7, 29.6, 29.4, 29.3, 29.2, 25.7, 25.6, 22.7, 14.1; EIMS (*m/z*): 403, 385, 294, 290, 265, 176, 163, 122, 104 (base), 91, 83, 69, 55, 43; HRMS–ESI: Calcd. for $C_{26}H_{45}NO_2$: 404.3529 $[M+H]^+$. Found: 404.3529.

References

1. Civitello, E. R.; Rapoport, H. *J. Org. Chem.* **1994**, *59*, 3775–3782.

2. Liu, R.; Ma, J.; Wei, B.; Lin, G.; Huang, W. *Tetrahedron Lett.* **2009**, *50*, 4046–4049.

3. Qabar, M. N.; Kahn, M. *Tetrahedron Lett.* **1996**, *37,* 965–968*.*

4. Sankaranarayanan, S.; Chattopadhyay, S. *Tetrahedron: Asymmetry* **1998**, *9*, 1345–1350.

5. Nanda, S.; Yadav, J. S. *Tetrahedron: Asymmetry* **2003**, *14*, 1799–1806.

^{1}H NMR and ^{13}C NMR spectra

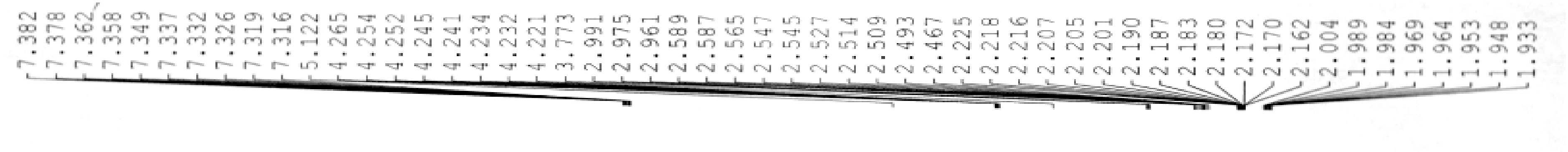

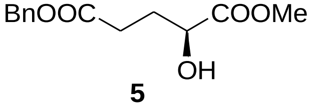

5

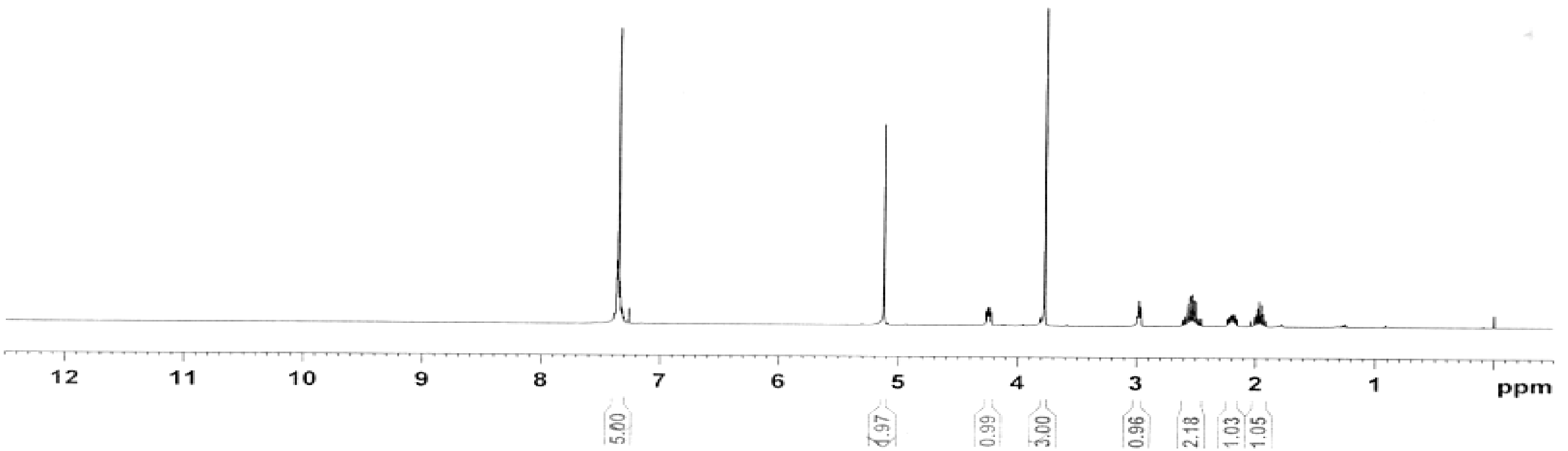

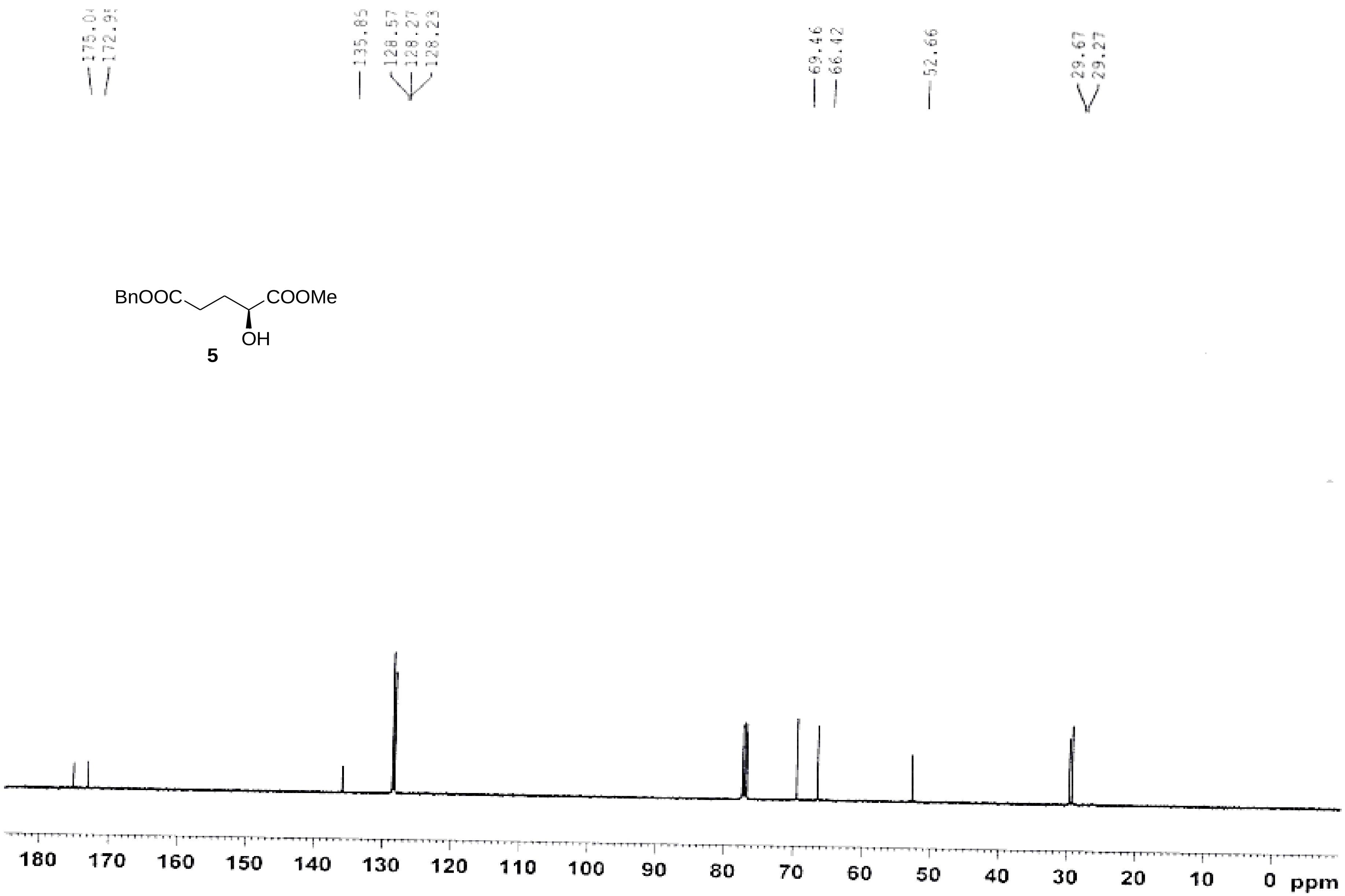
175.04
172.95
135.85
128.57
128.27
128.23
69.46
66.42
52.66
29.67
29.27
BnOOC
COOMe
OH
5
180 170 160 150 140 130 120 110 100 90 80 70 60 50 40 30 20 10 0 ppm

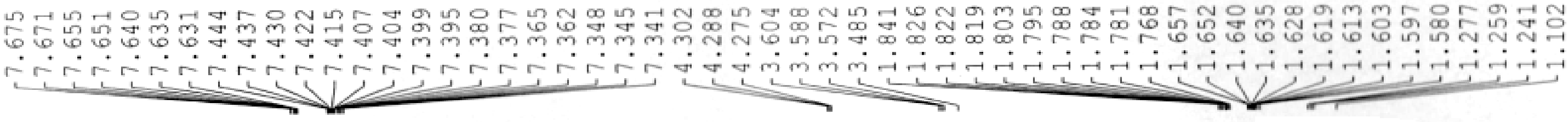
7.675
7.671
7.655
7.651
7.640
7.635
7.631
7.444
7.437
7.430
7.422
7.415
7.407
7.404
7.399
7.395
7.380
7.377
7.365
7.362
7.348
7.345
7.341
4.302
4.288
4.275
3.604
3.588
3.572
3.485
1.841
1.826
1.822
1.819
1.803
1.795
1.788
1.784
1.781
1.768
1.657
1.652
1.640
1.635
1.628
1.619
1.613
1.603
1.597
1.580
1.277
1.259
1.241
1.102

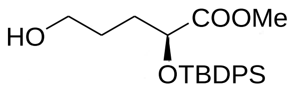
HO
COOMe
OTBDPS

7

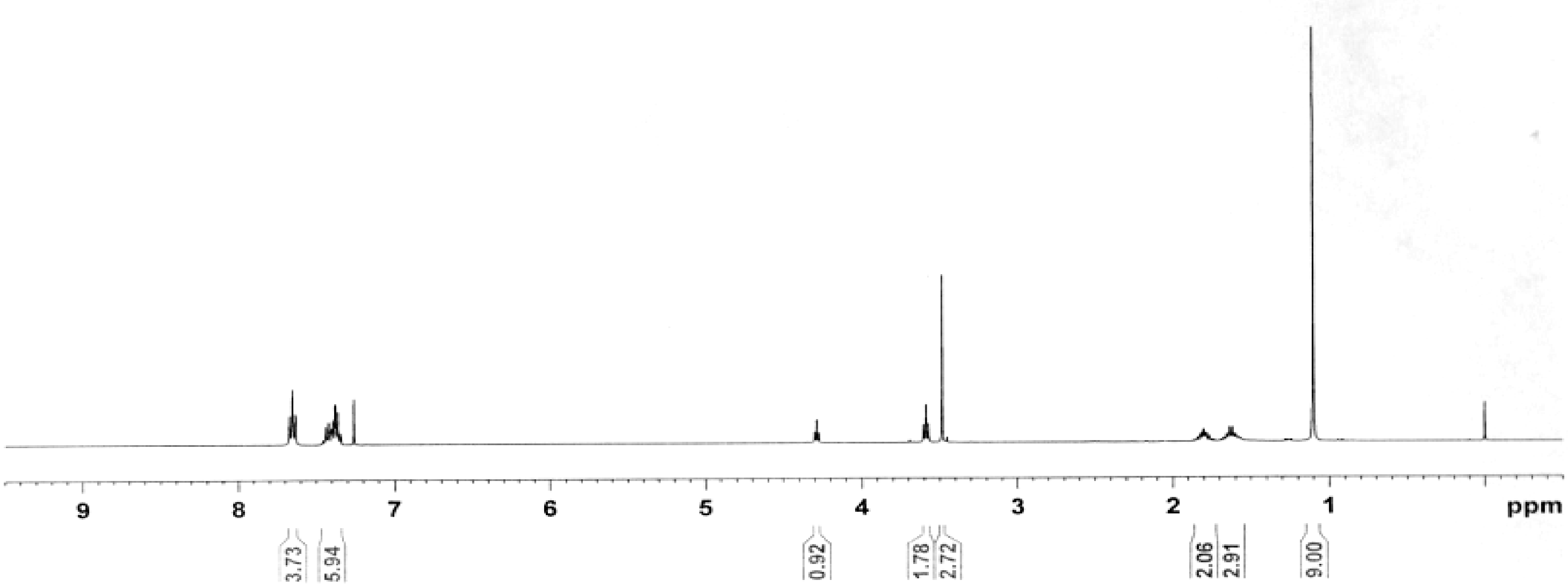
9
8
7
6
5
4
3
2
1
ppm
3.73
5.94
0.92
1.78
2.72
2.06
2.91
9.00

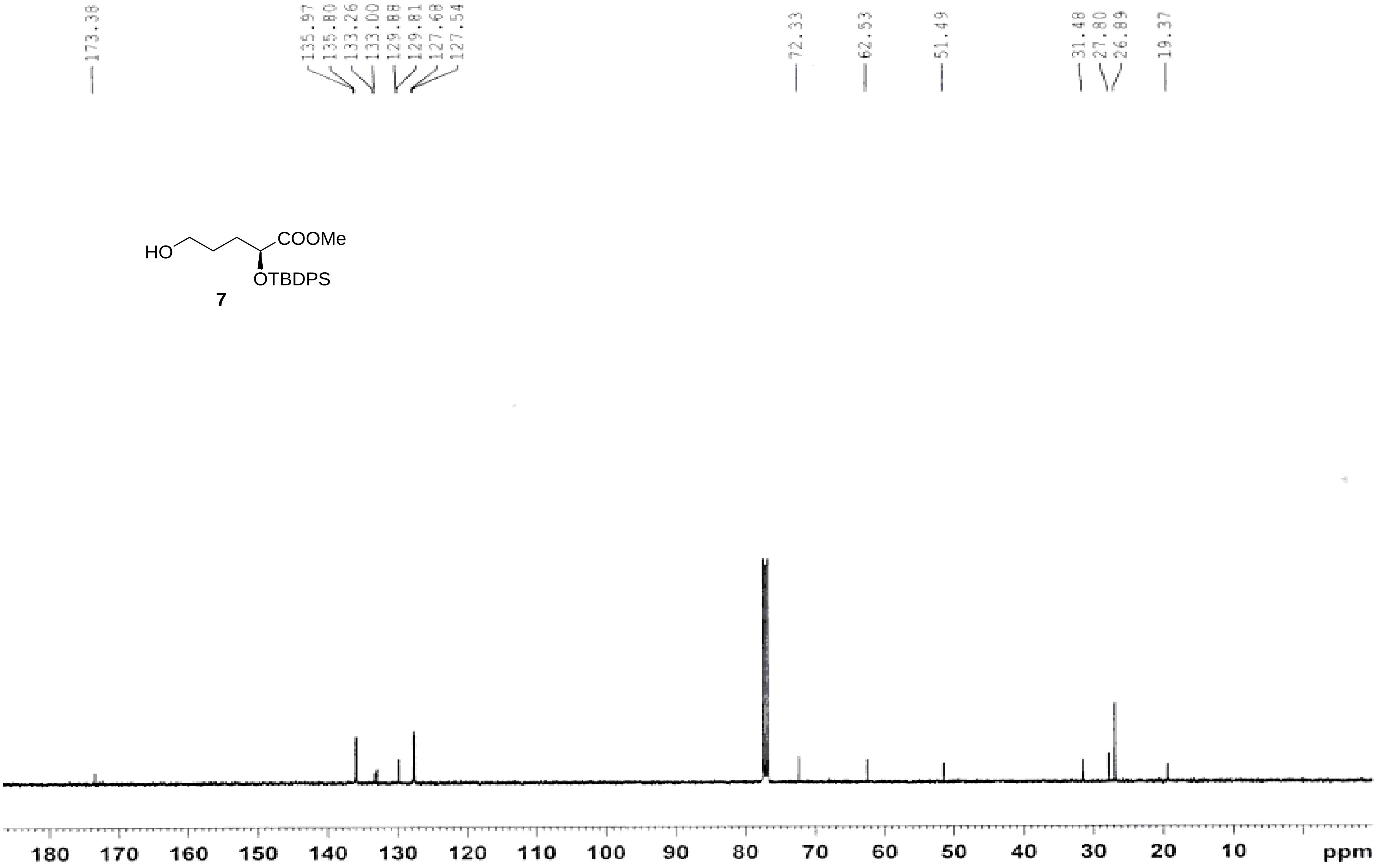
173.38
135.97
135.80
133.26
133.00
129.88
129.81
127.68
127.54
72.33
62.53
51.49
31.48
27.80
26.89
19.37
180 170 160 150 140 130 120 110 100 90 80 70 60 50 40 30 20 10 ppm
HO
COOMe
OTBDPS

7

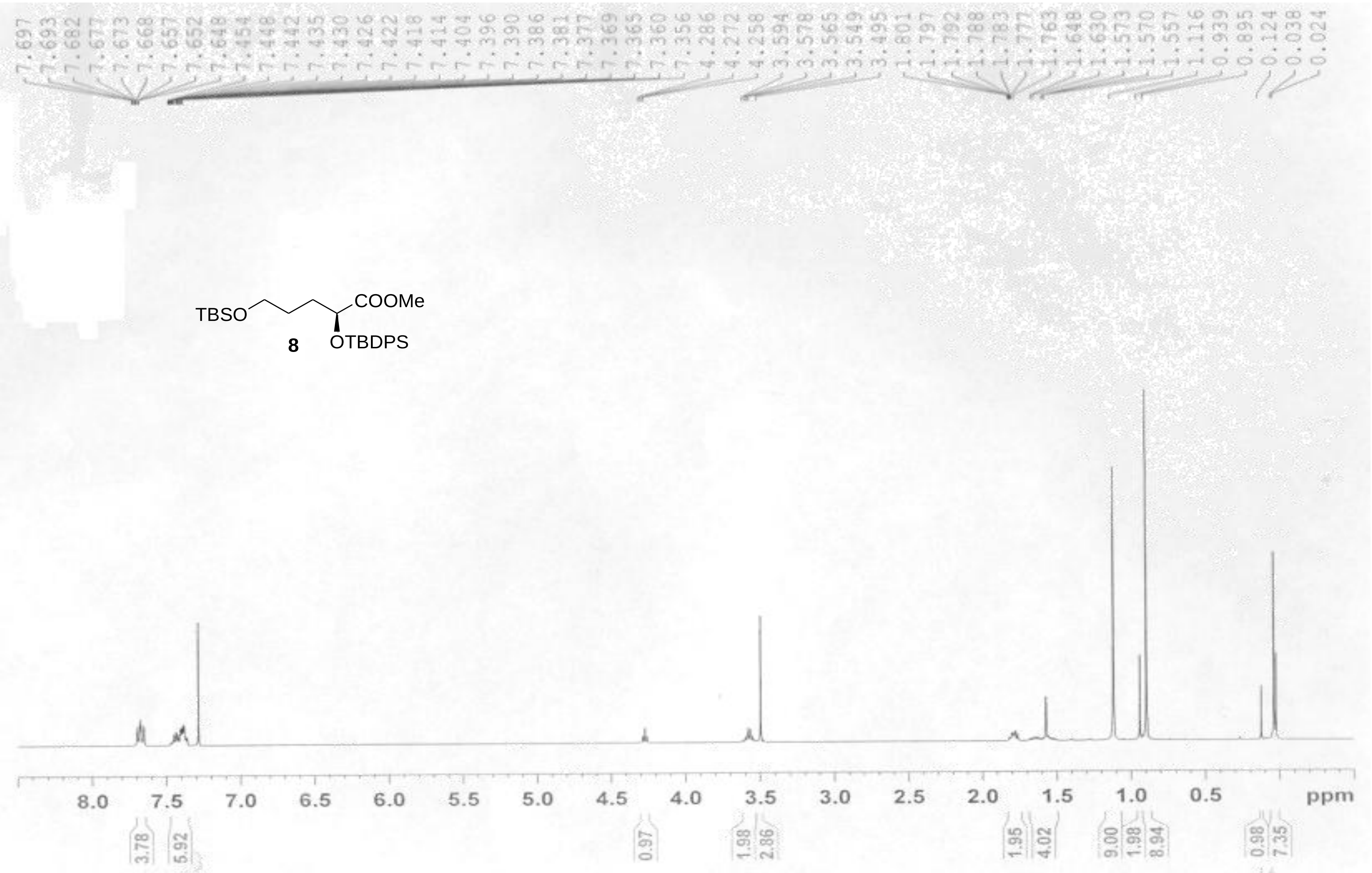
TBSO
COOMe
8
OTBDPS
ppm

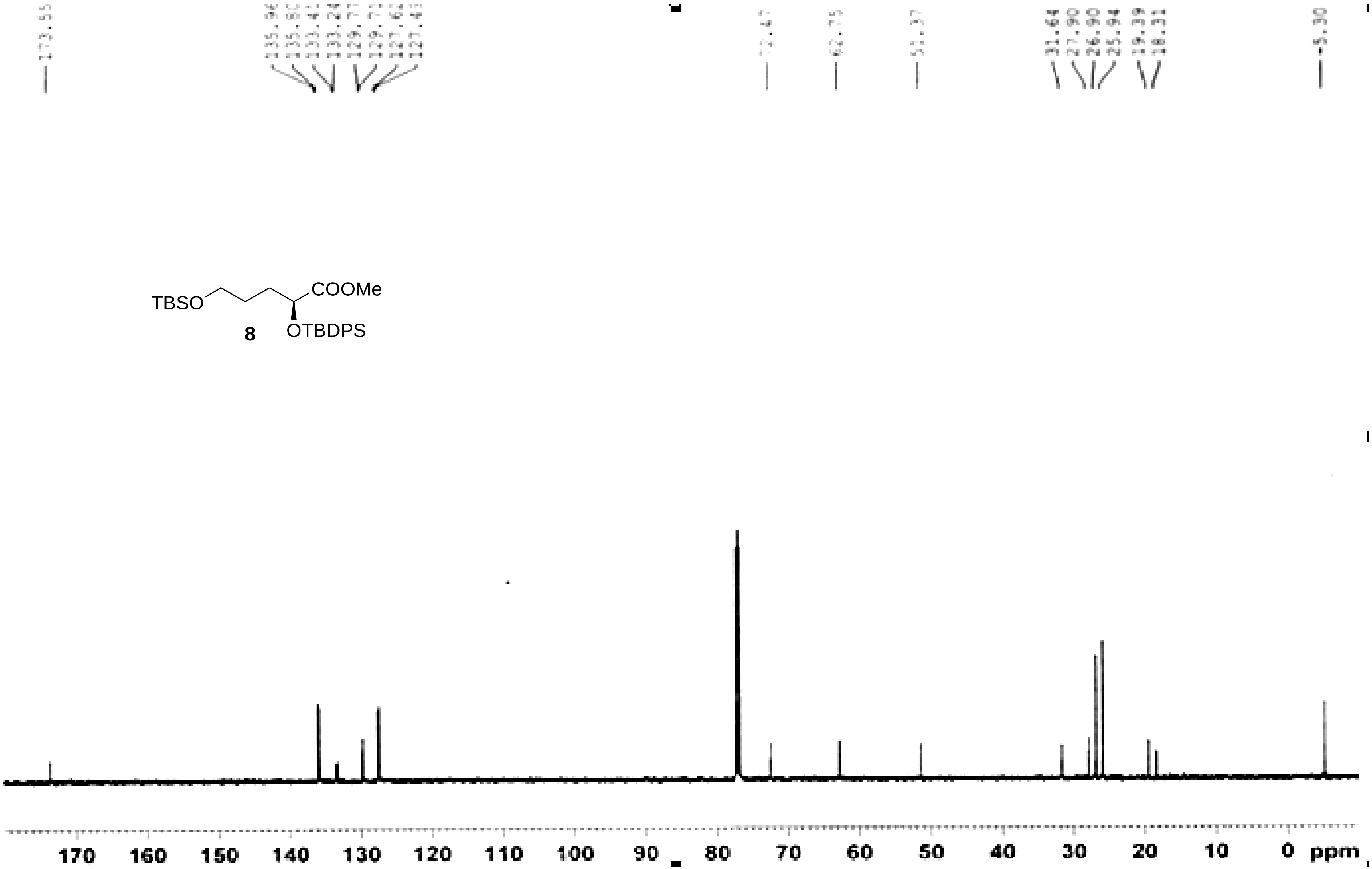
173.55
62.75
51.37
31.64
27.90
26.90
25.94
19.39
18.31
-5.30
TBSO
COOMe
8
OTBDPS
170
160
150
140
130
120
110
100
90
80
70
60
50
40
30
20
10
0
ppm

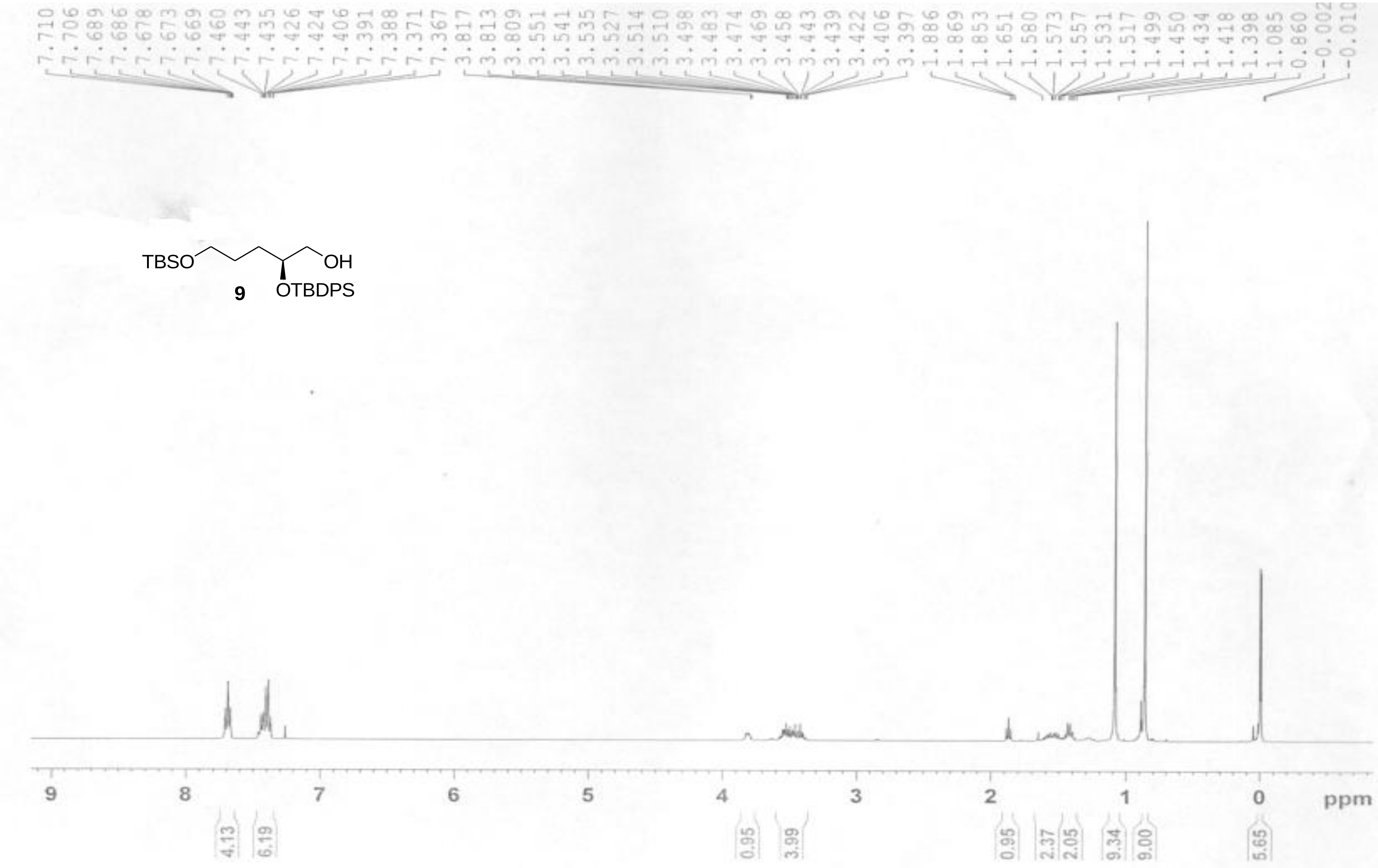
TBSO
OH
9
OTBDPS
ppm

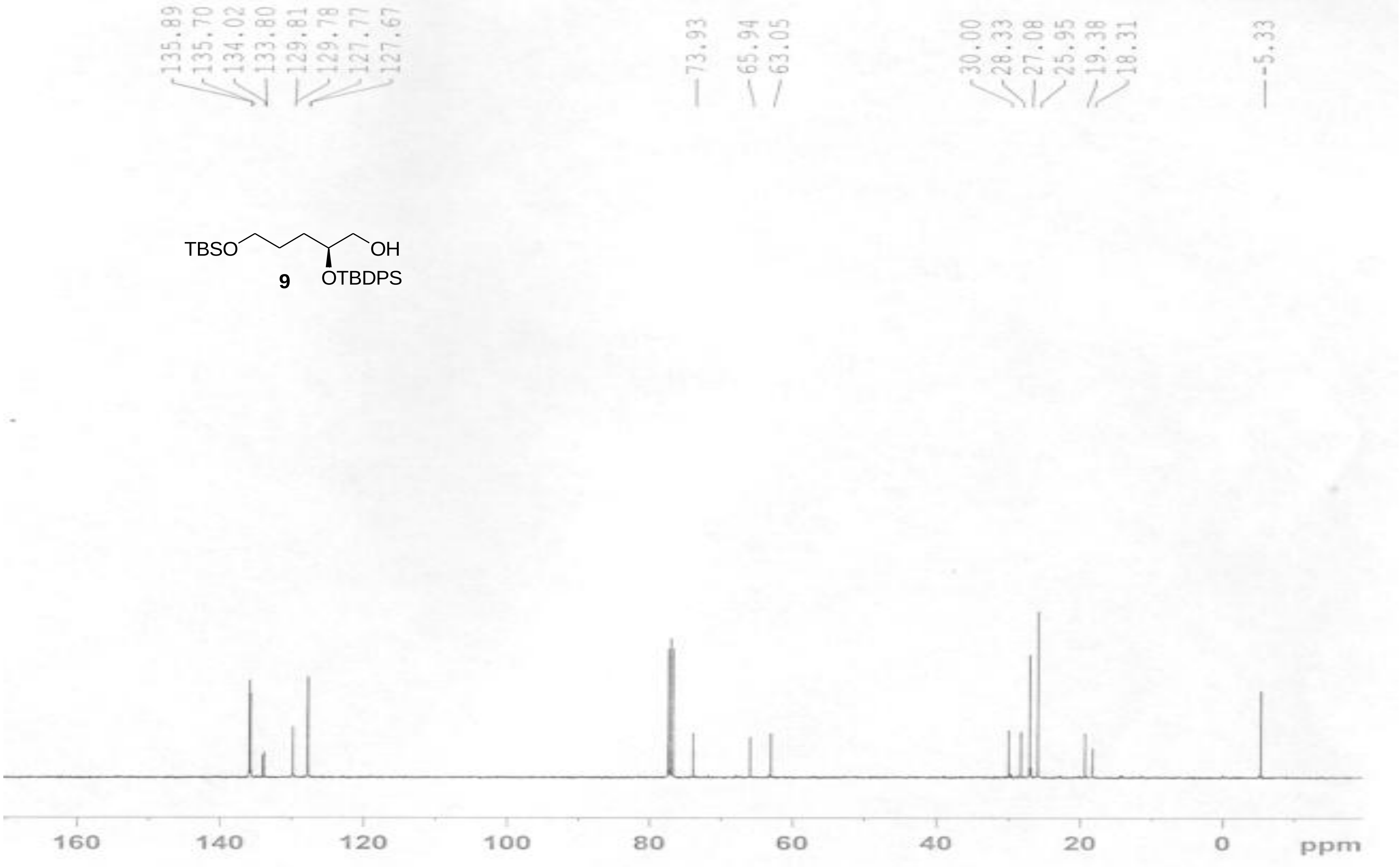
135.89
135.70
134.02
133.80
129.81
129.78
127.77
127.67
73.93
65.94
63.05
30.00
28.33
27.08
25.95
19.38
18.31
-5.33
TBSO
OH
OTBDPS
9
160
140
120
100
80
60
40
20
0
ppm

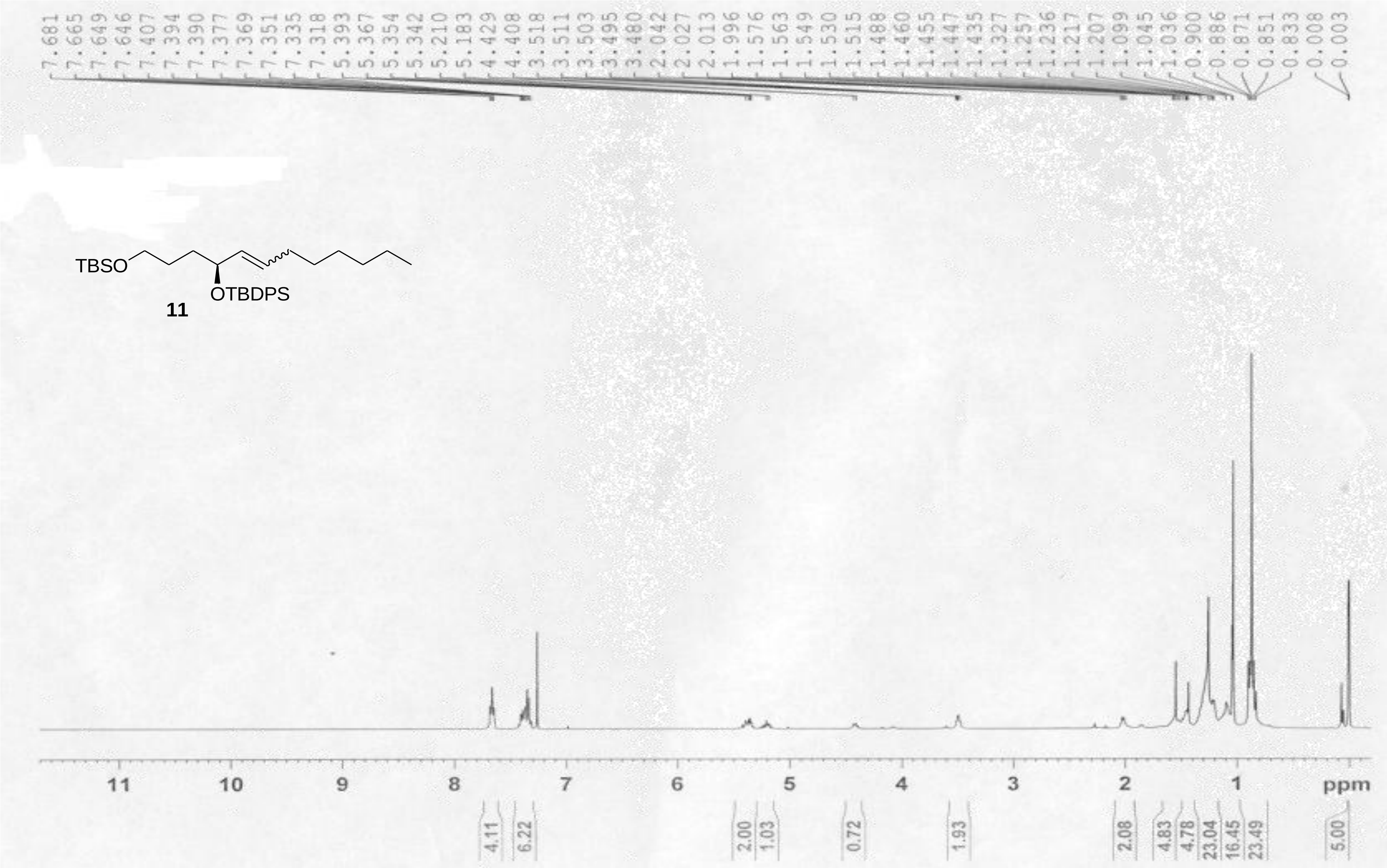

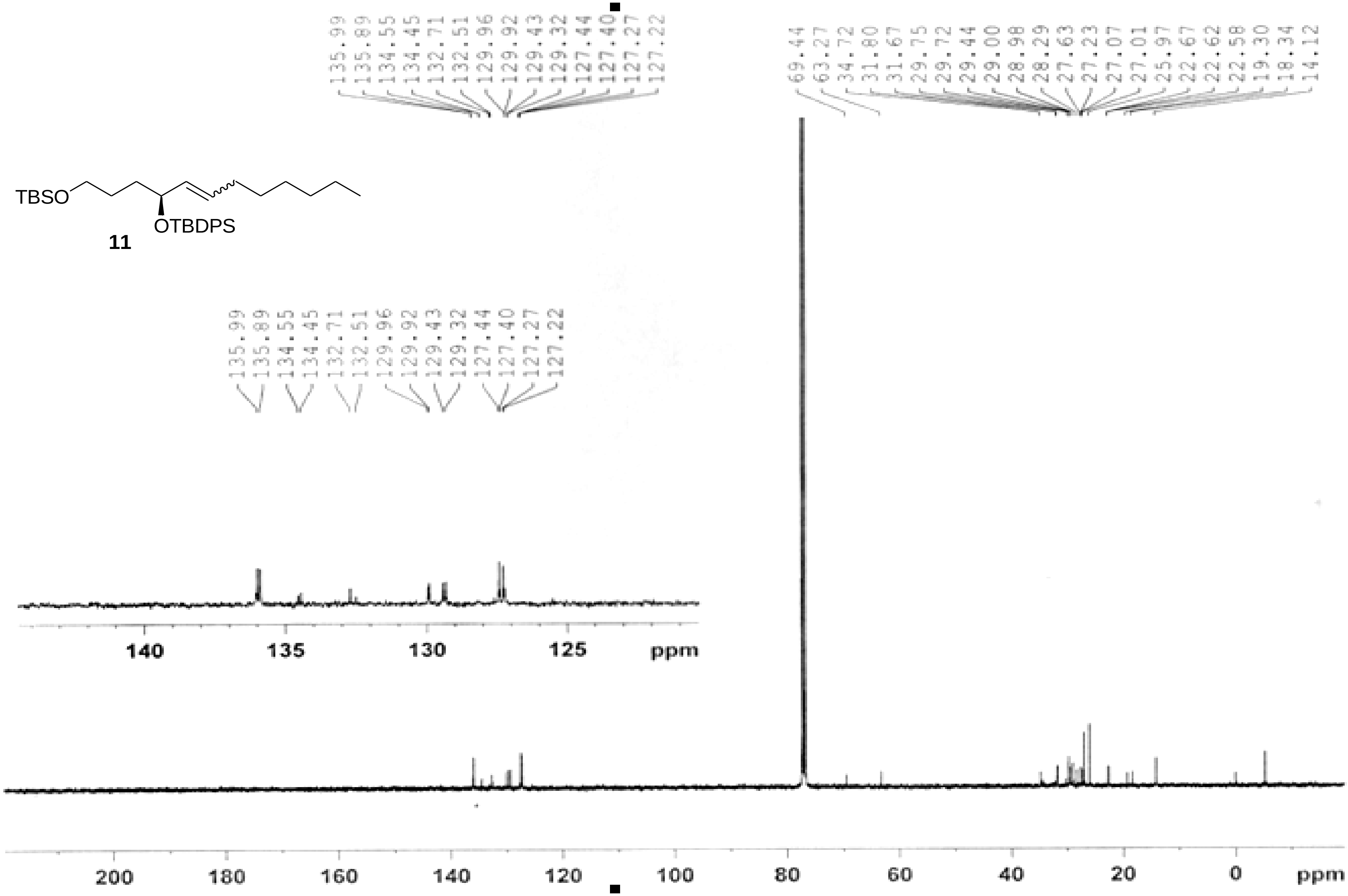
135.99 135.89 134.55 134.45 132.71 132.51 129.96 129.92 129.43 129.32 127.44 127.40 127.27 127.22
69.44 63.27 34.72 31.80 31.67 29.75 29.72 29.44 29.00 28.98 28.29 27.63 27.23 27.07 27.01 25.97 22.67 22.62 22.58 19.30 18.34 14.12
TBSO
OTBDPS
11
135.99 135.89 134.55 134.45 132.71 132.51 129.96 129.92 129.43 129.32 127.44 127.40 127.27 127.22
140 135 130 125 ppm
200 180 160 140 120 100 80 60 40 20 0 ppm

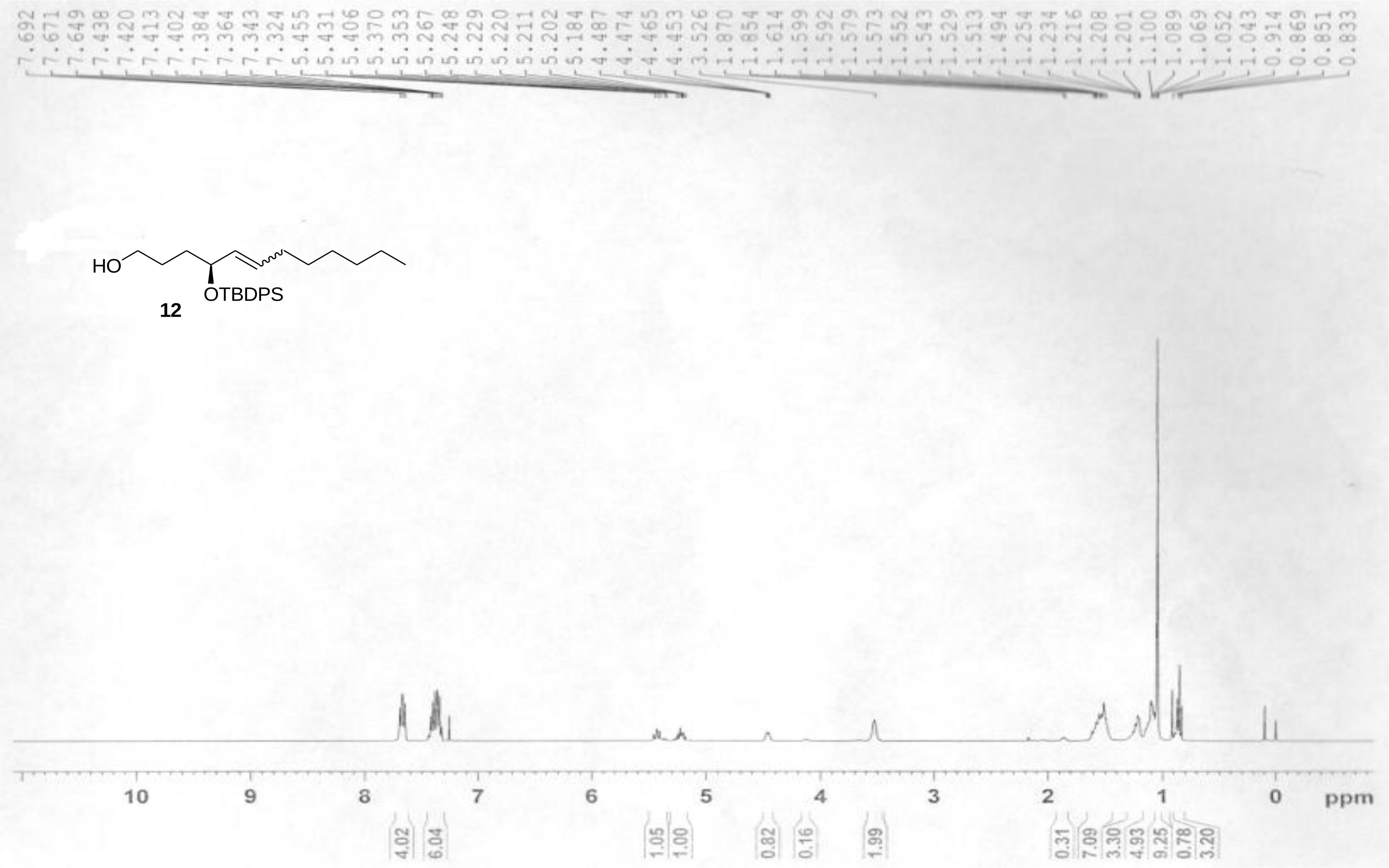
HO
OTBDPS
12
ppm

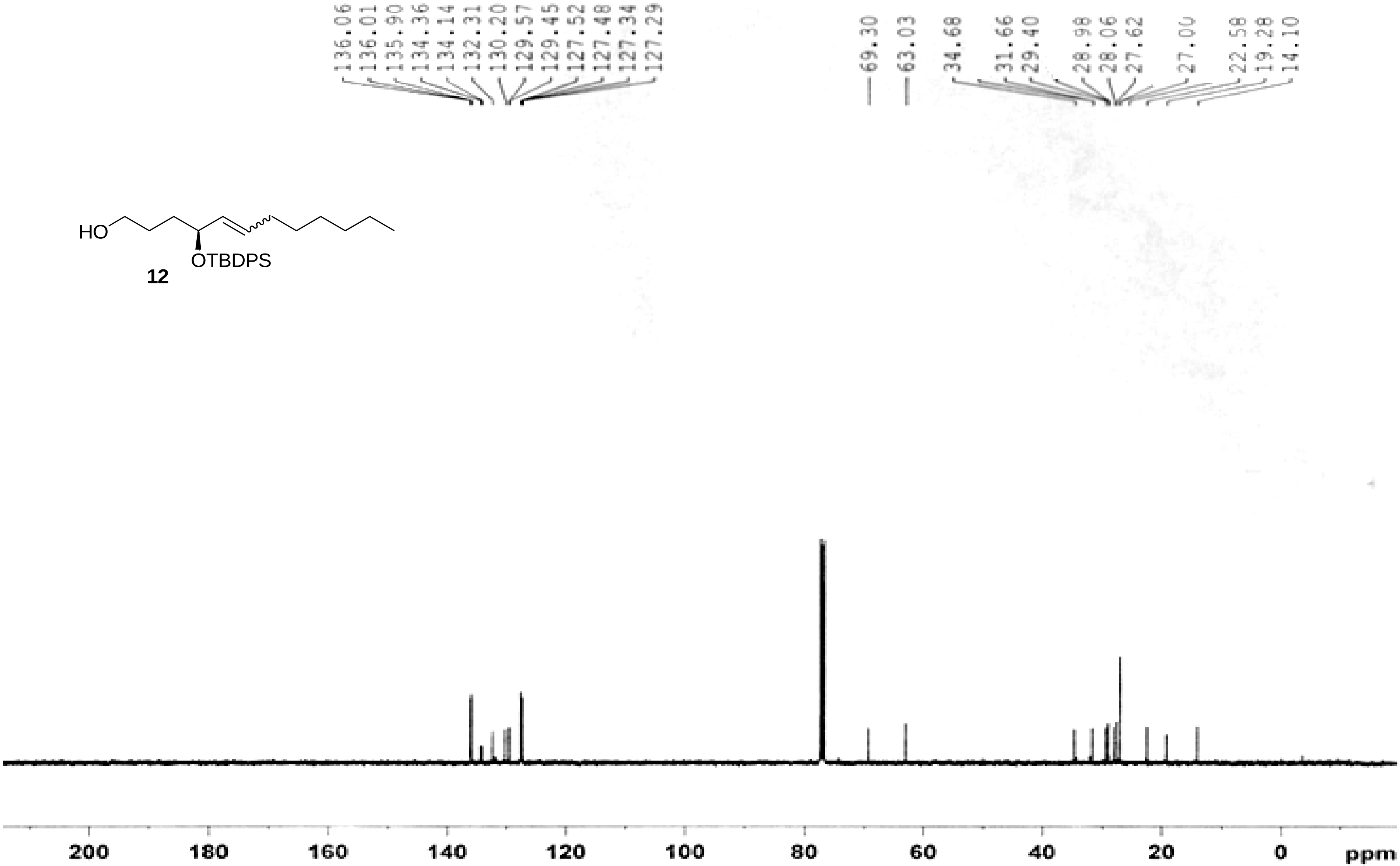
HO
OTBDPS
12
136.06
136.01
135.90
134.36
134.14
132.31
130.20
129.57
129.45
127.52
127.48
127.34
127.29
69.30
63.03
34.68
31.66
29.40
28.98
28.06
27.62
27.00
22.58
19.28
14.10
200
180
160
140
120
100
80
60
40
20
0
ppm

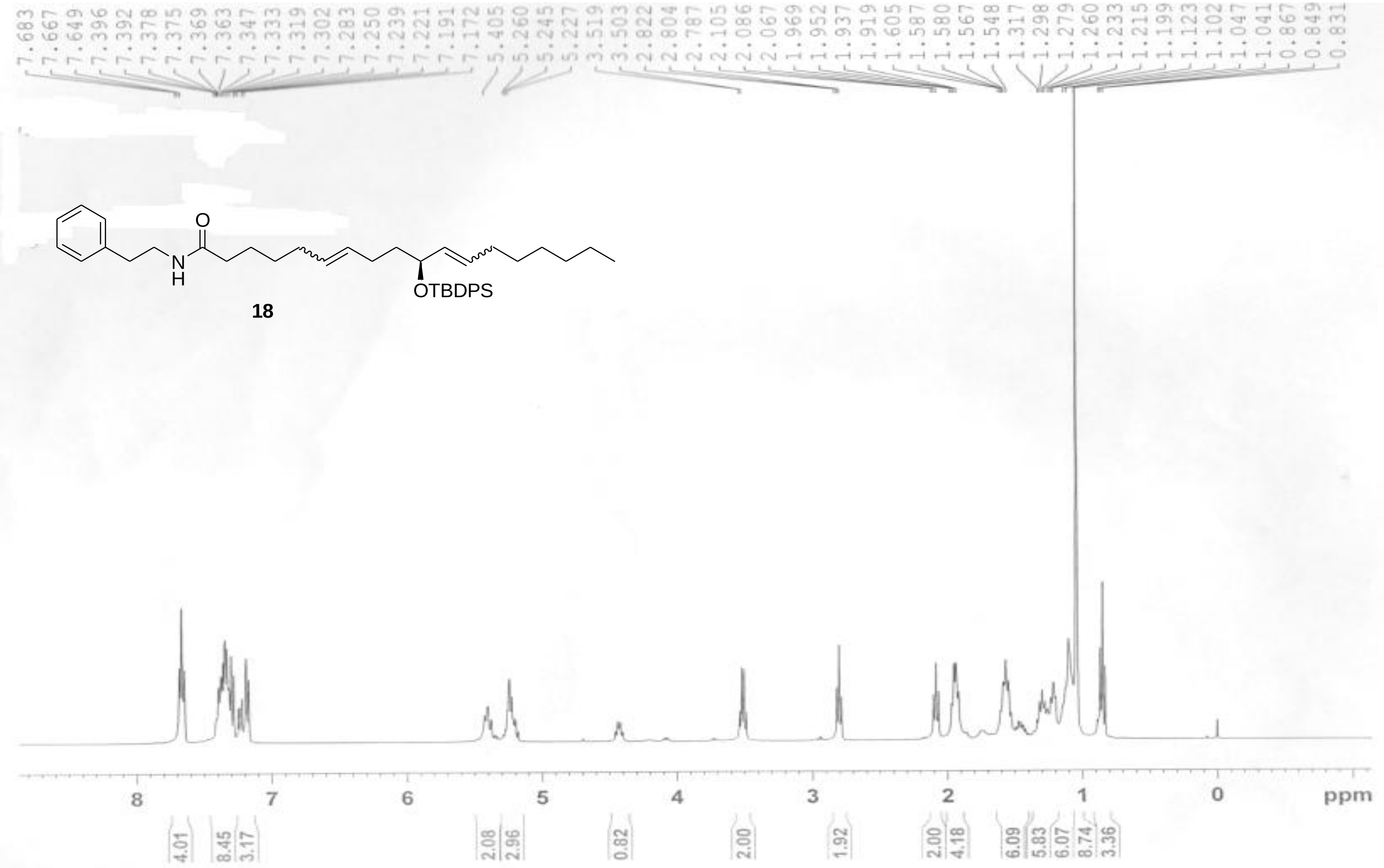

18

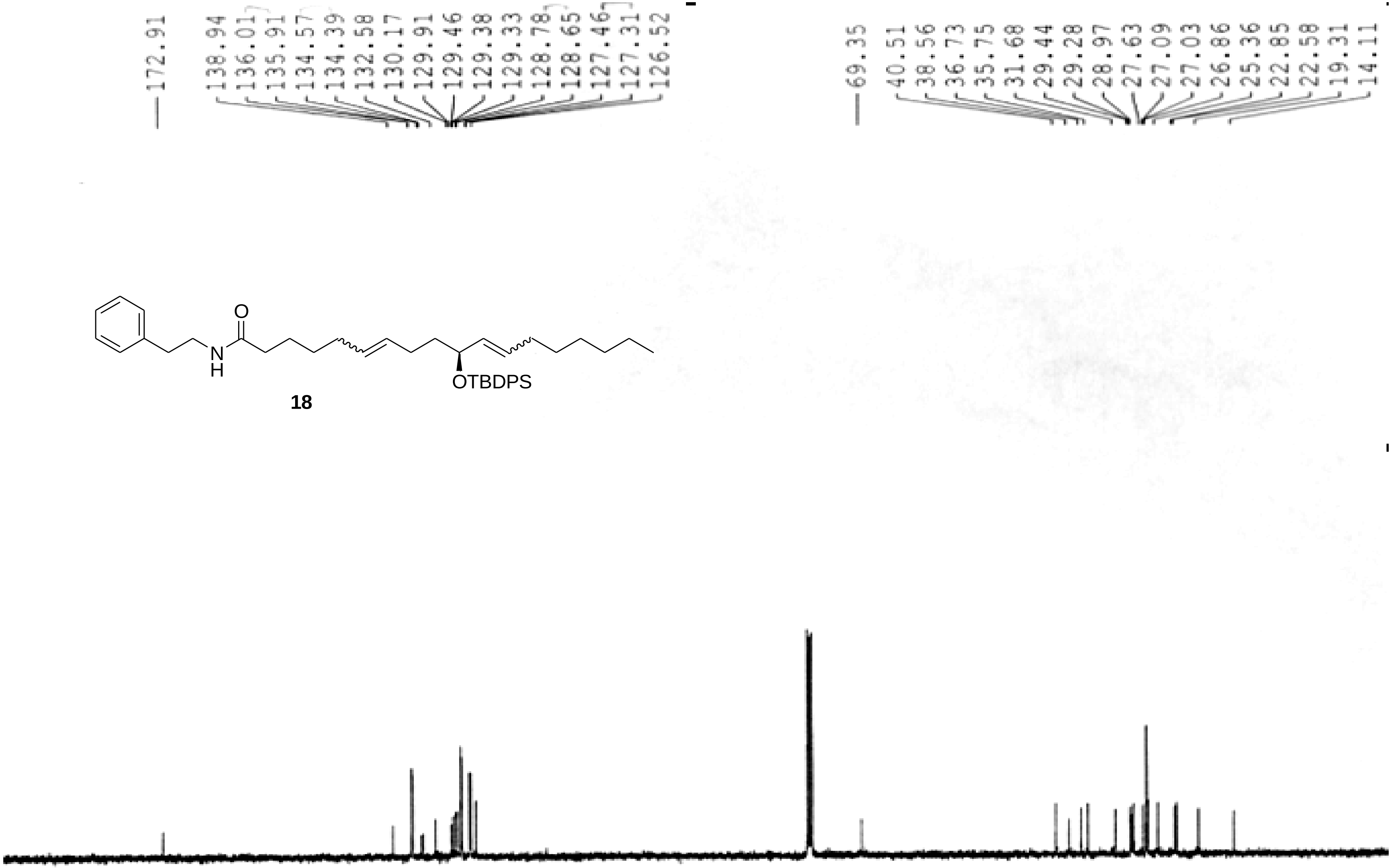
172.91
138.94
136.01
135.91
134.57
134.39
132.58
130.17
129.91
129.46
129.38
129.33
128.78
128.65
127.46
127.31
126.52
69.35
40.51
38.56
36.73
35.75
31.68
29.44
29.28
28.97
27.63
27.09
27.03
26.86
25.36
22.85
22.58
19.31
14.11
O
N
H
OTBDPS
18

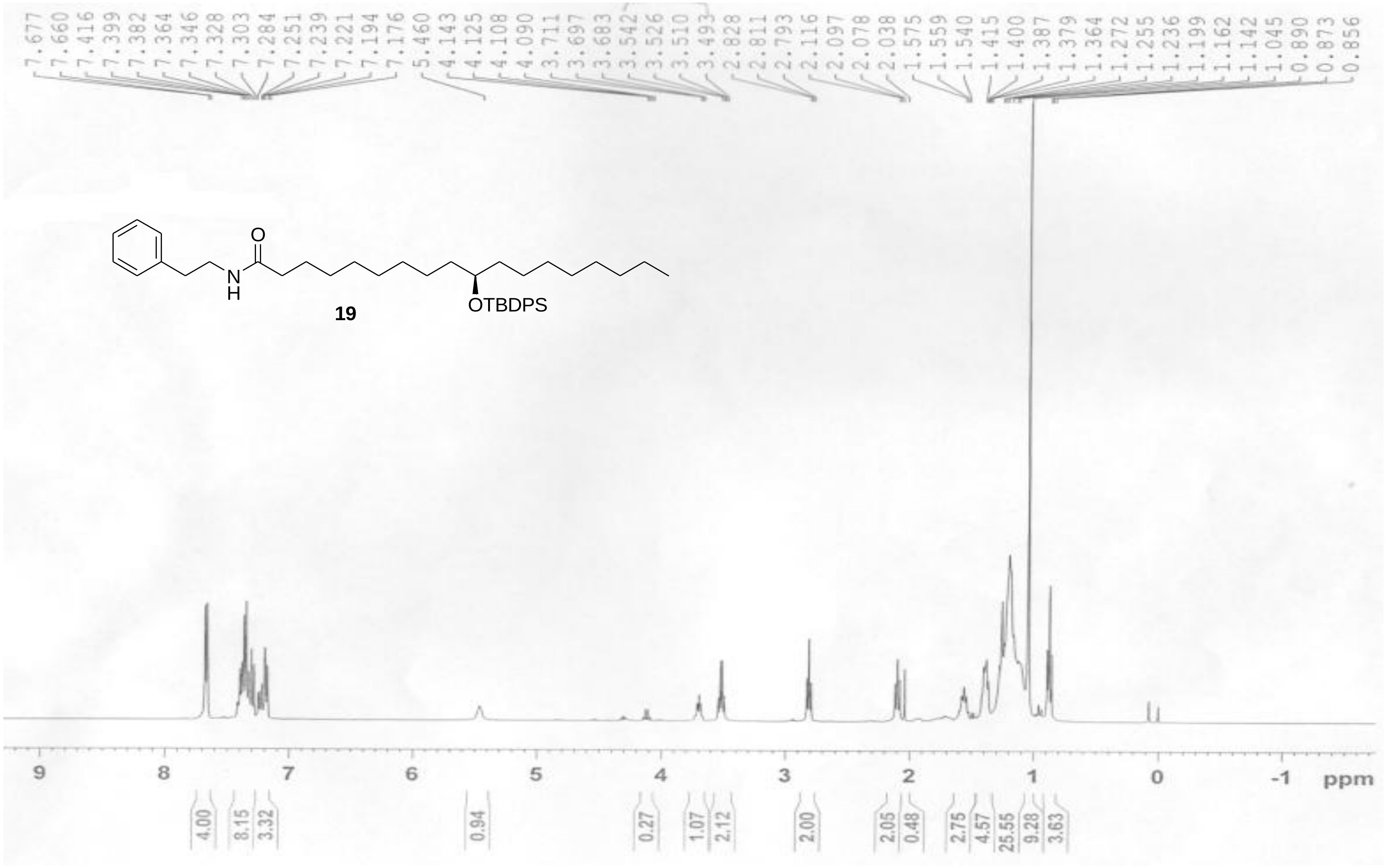
OTBDPS
19
ppm

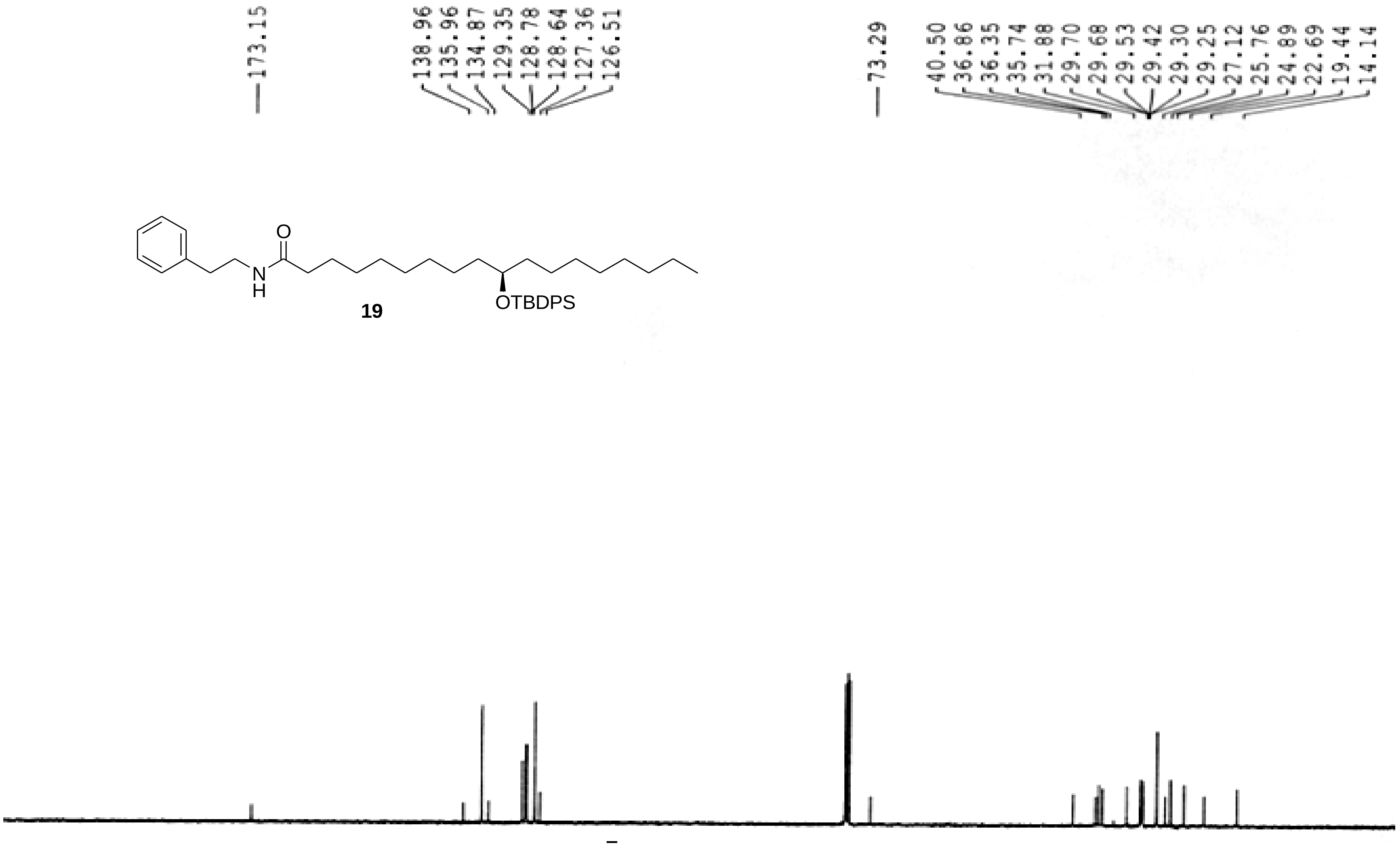
173.15
138.96
135.96
134.87
129.35
128.78
128.64
127.36
126.51
73.29
40.50
36.86
36.35
35.74
31.88
29.70
29.68
29.53
29.42
29.30
29.25
27.12
25.76
24.89
22.69
19.44
14.14
O
N
H
OTBDPS
19

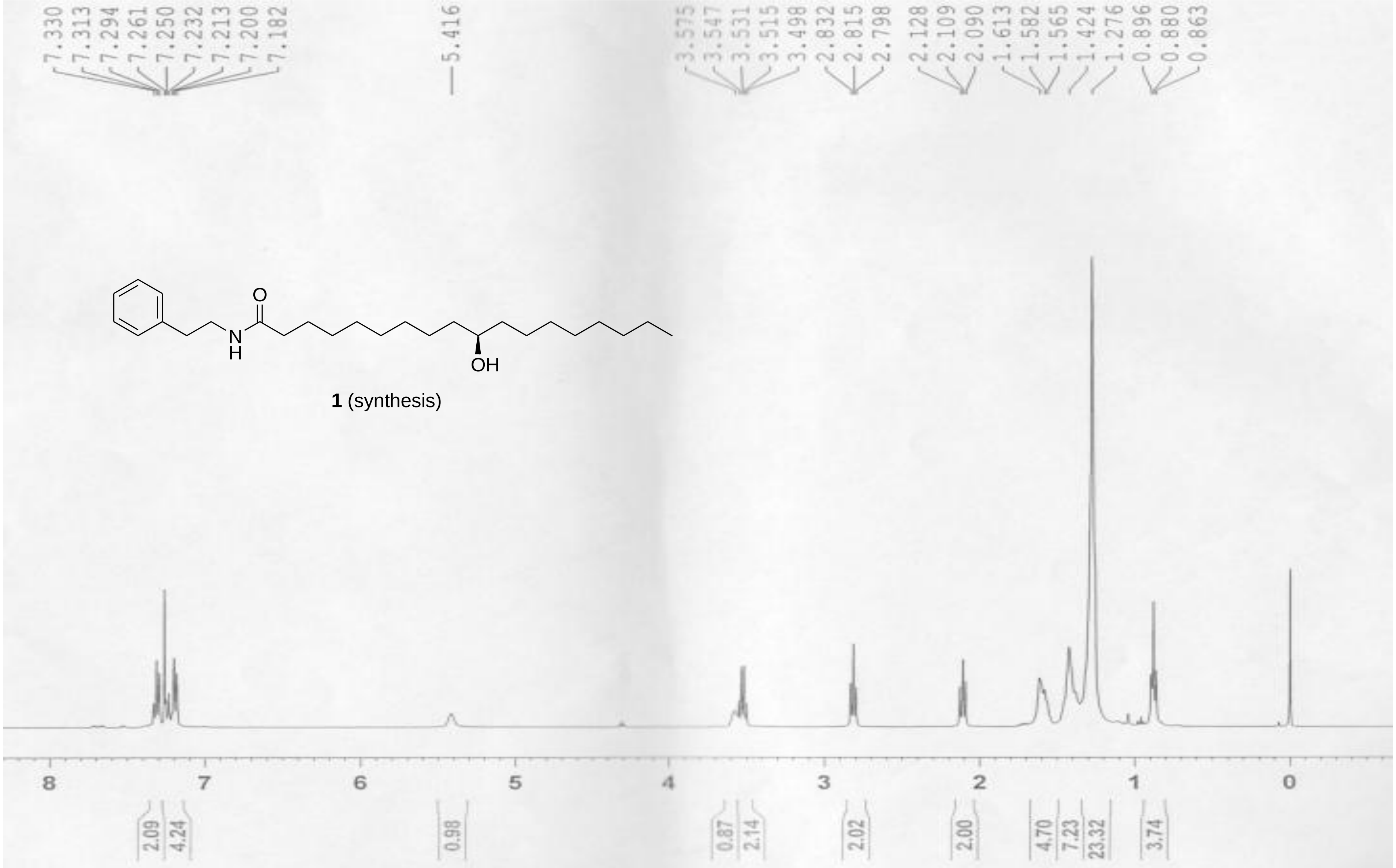
7.330
7.313
7.294
7.261
7.250
7.232
7.213
7.200
7.182
5.416
3.575
3.547
3.531
3.515
3.498
2.832
2.815
2.798
2.128
2.109
2.090
1.613
1.582
1.565
1.424
1.276
0.896
0.880
0.863
O
N
H
OH
8
7
6
5
4
3
2
1
0
2.09
4.24
0.98
0.87
2.14
2.02
2.00
4.70
7.23
23.32
3.74

1 (synthesis)

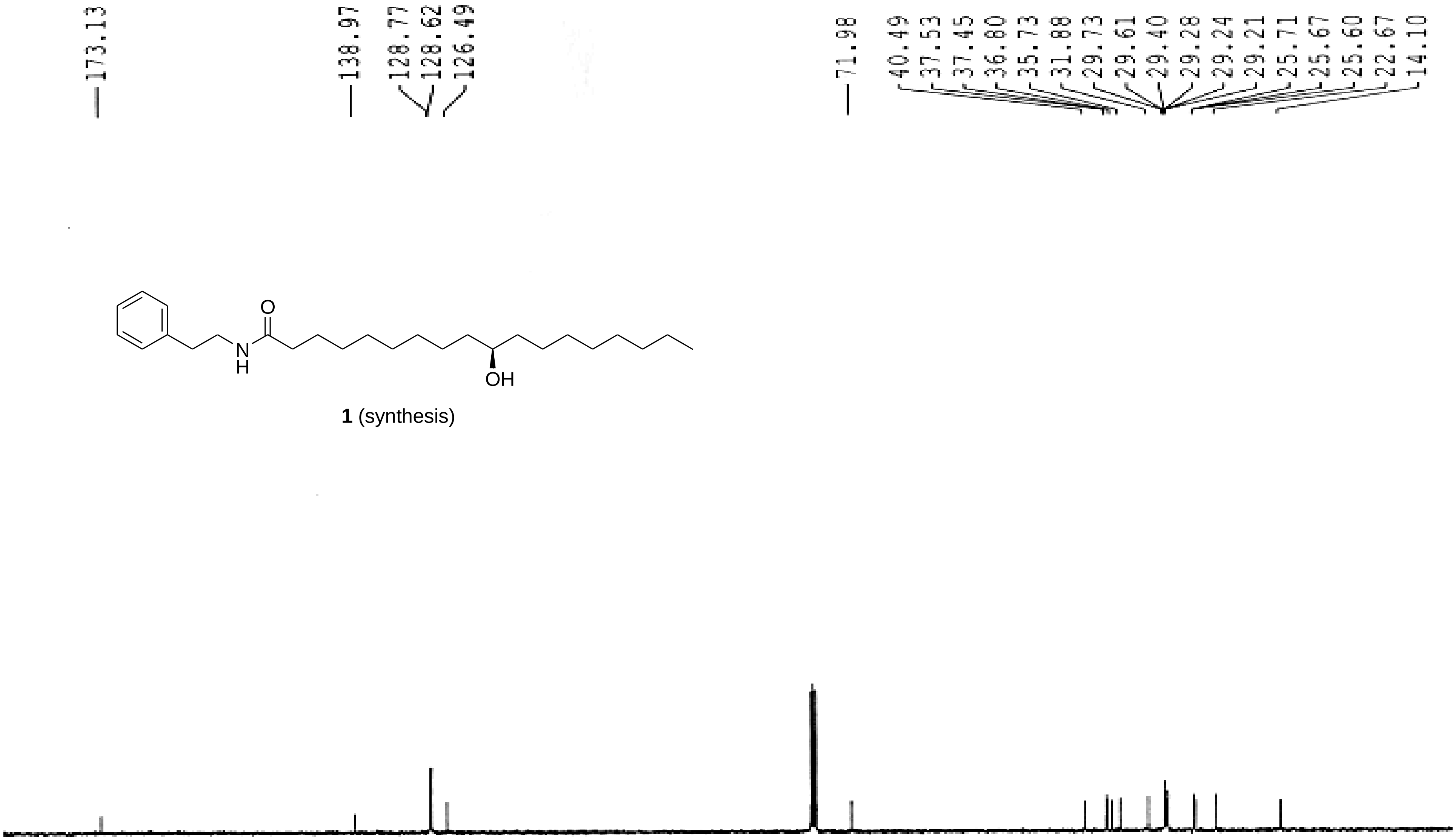
173.13
138.97
128.77
128.62
126.49
71.98
40.49
37.53
37.45
36.80
35.73
31.88
29.73
29.61
29.40
29.28
29.24
29.21
25.71
25.67
25.60
22.67
14.10
O
N
H
OH

1 (synthesis)

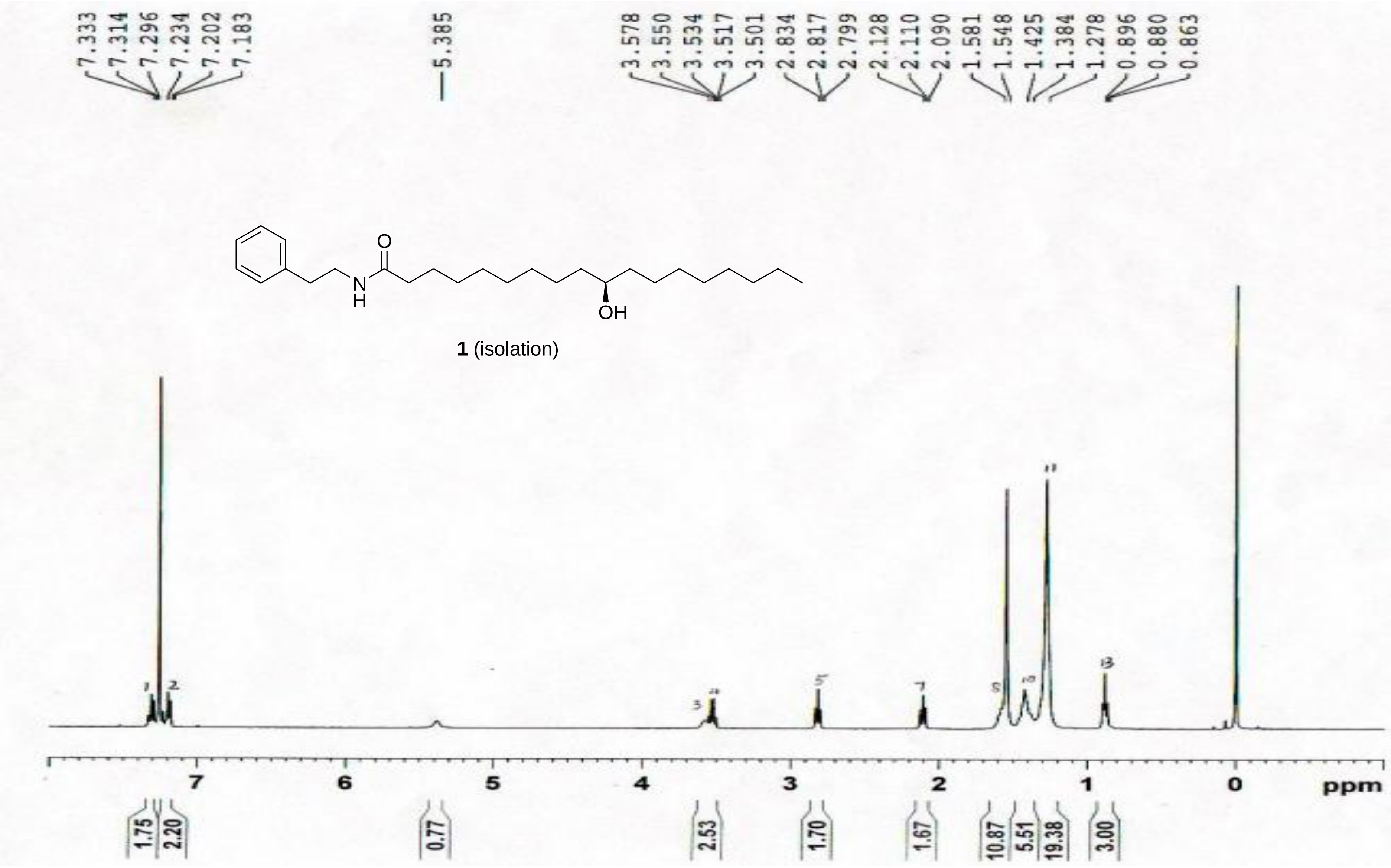

1 (isolation)

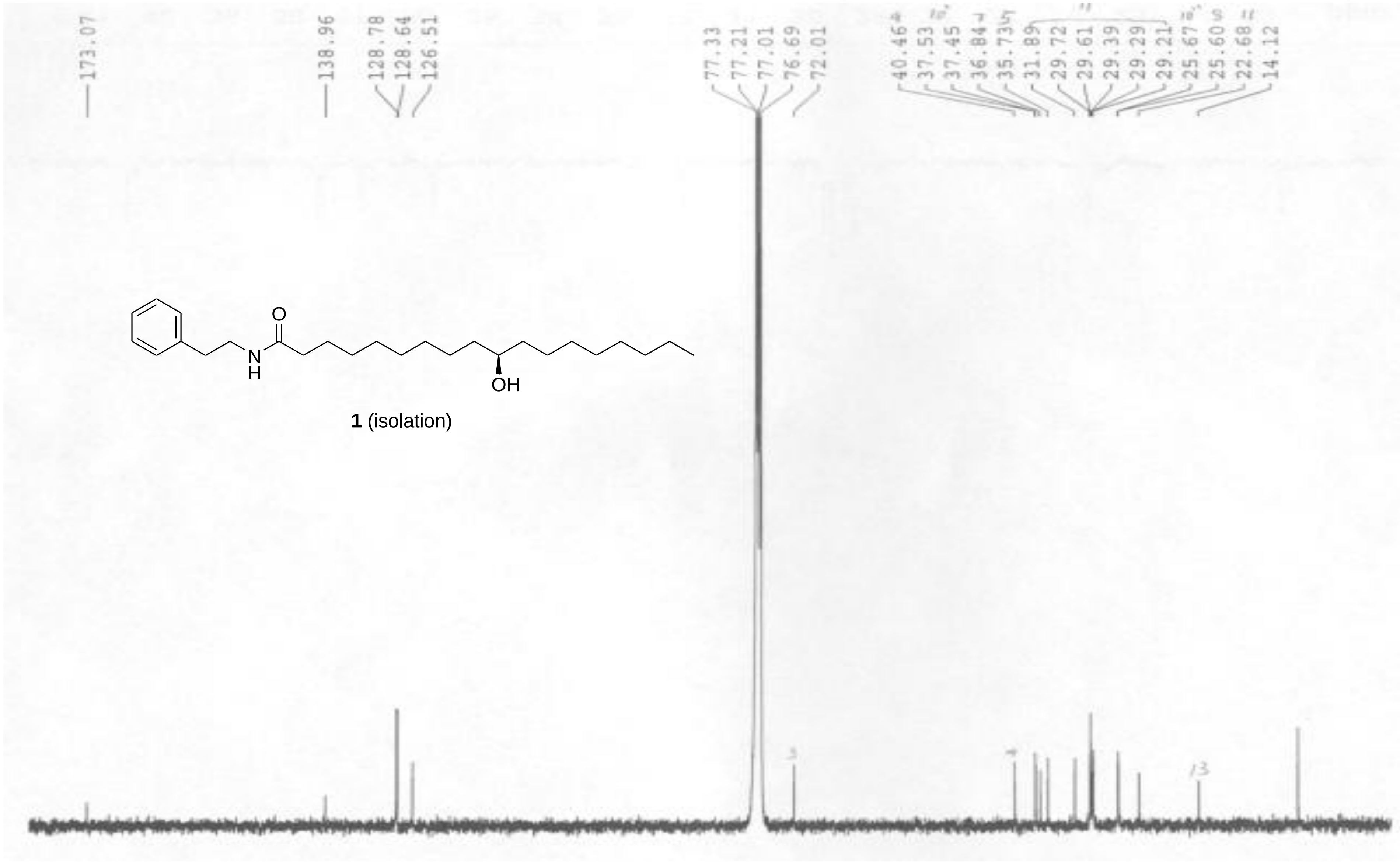
173.07
138.96
128.78
128.64
126.51
77.33
77.21
77.01
76.69
72.01
40.46
37.53
37.45
36.84
35.73
31.89
29.72
29.61
29.39
29.29
29.21
25.67
25.60
22.68
14.12
O
N
H
OH

1 (isolation)

HMQC of compound **1**

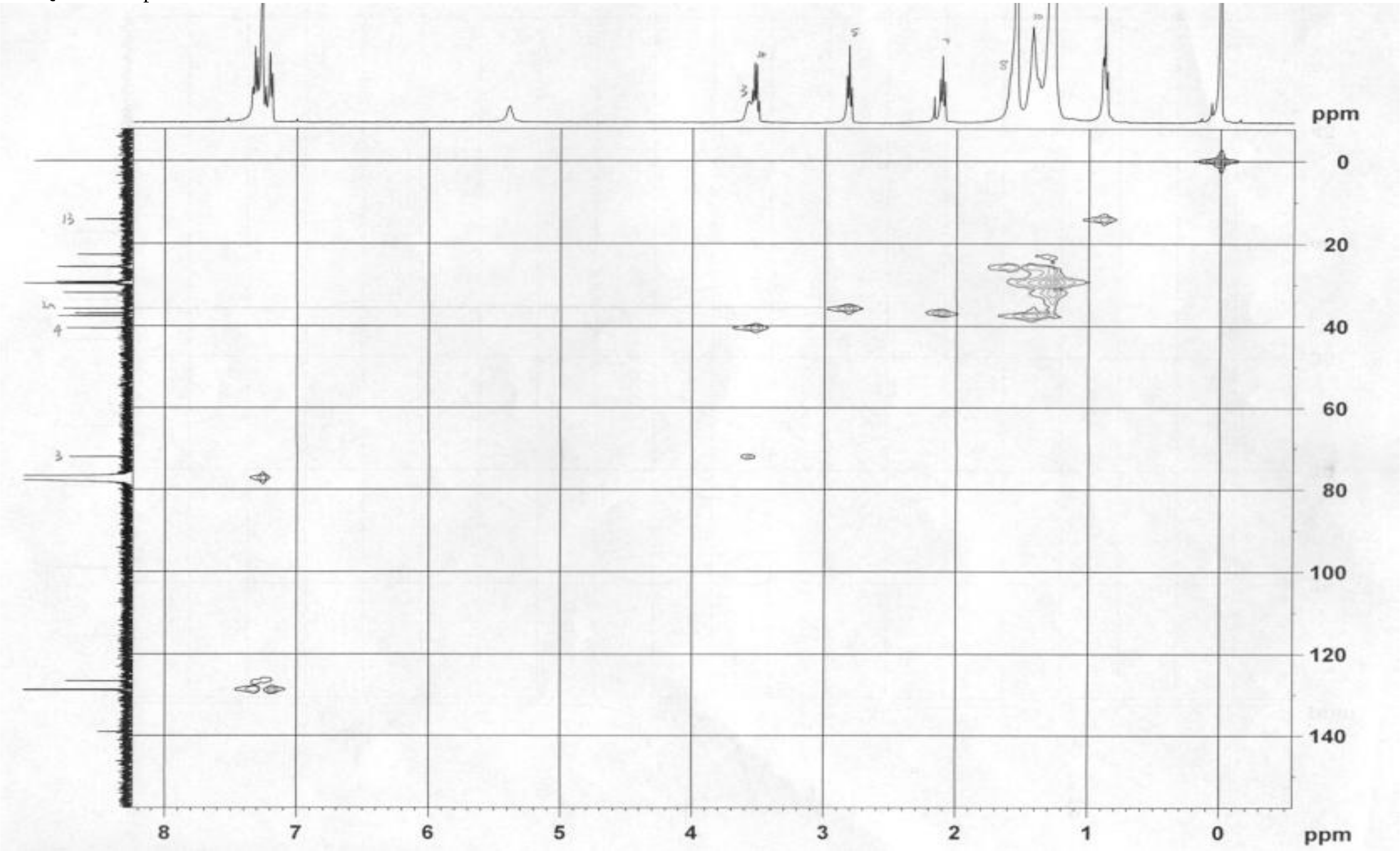

COSY of compound **1**

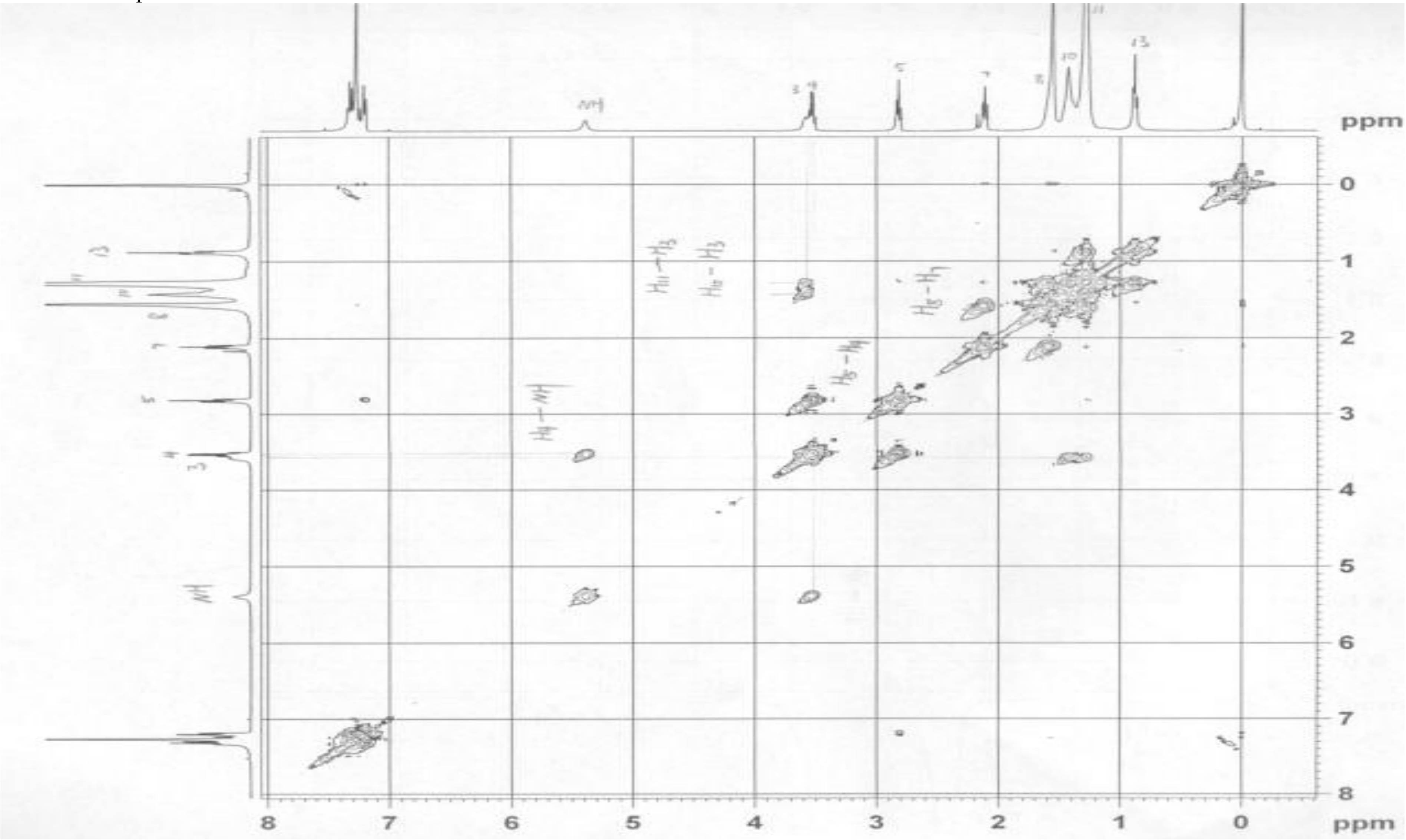

HMBC of compound **1**

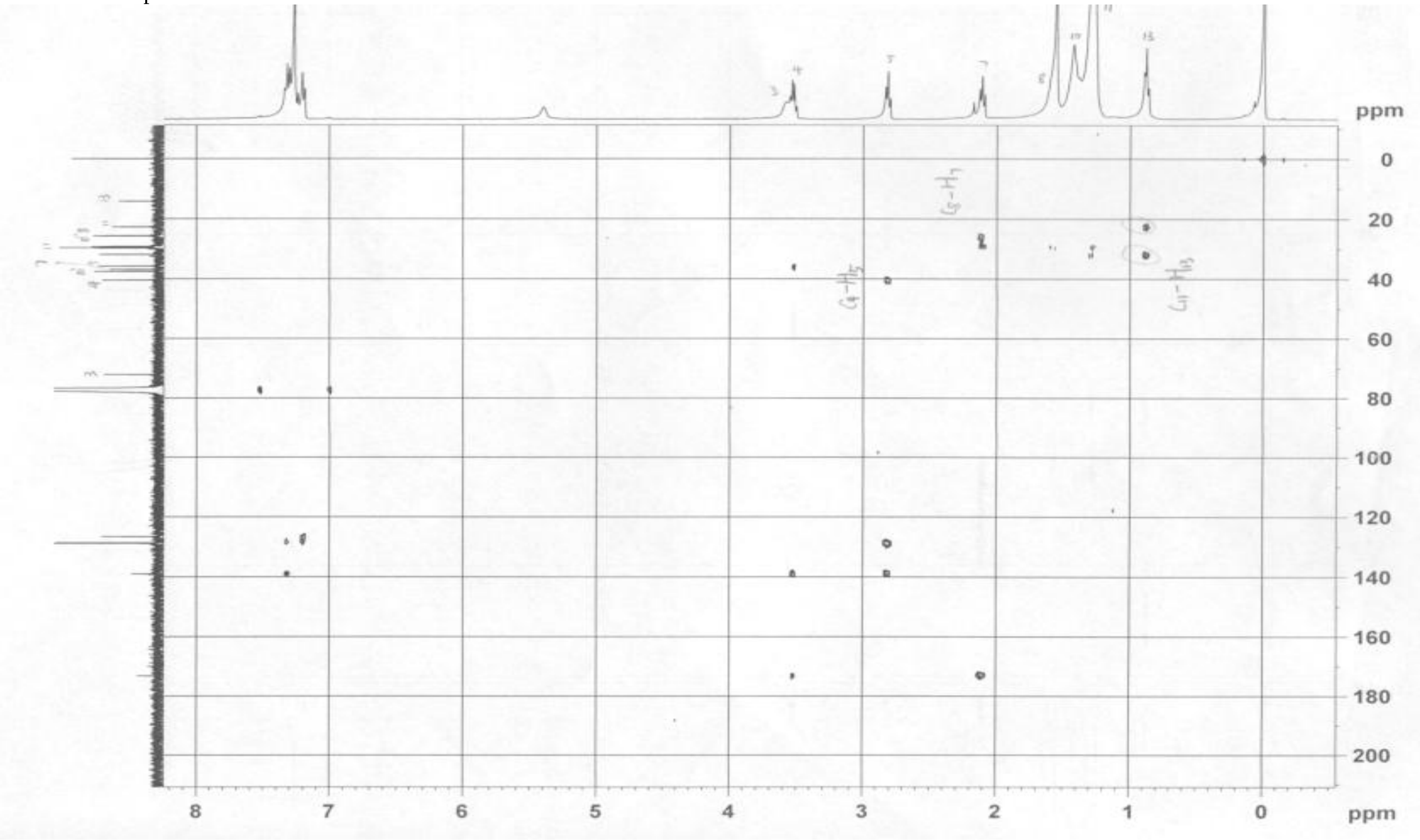

Supplement: File 1 — Detailed experimental procedures for the synthesis of compound 1. [file Beilstein_J_Org_Chem-07-1342-s001.pdf]
